# Dual-Channel Fluorescent Probe for the Simultaneous Monitoring of Peroxynitrite and Adenosine-5′-triphosphate in Cellular Applications

**DOI:** 10.1021/jacs.1c07954

**Published:** 2021-12-21

**Authors:** Luling Wu, Jihong Liu, Xue Tian, Robin R. Groleau, Beidou Feng, Yonggang Yang, Adam C. Sedgwick, Hai-Hao Han, Yang Wang, Han-Min Wang, Fang Huang, Steven D. Bull, Hua Zhang, Chusen Huang, Yi Zang, Jia Li, Xiao-Peng He, Ping Li, Bo Tang, Tony D. James, Jonathan L. Sessler

**Affiliations:** † College of Chemistry, Chemical Engineering and Materials Science, Key Laboratory of Molecular and Nano Probes, Ministry of Education, Collaborative Innovation Center of Functionalized Probes for Chemical Imaging in Universities of Shandong, Institutes of Biomedical Sciences, Shandong Normal University, Jinan 250014, People’s Republic of China; □ The Education Ministry Key Laboratory of Resource Chemistry, Shanghai Key Laboratory of Rare Earth Functional Materials, and Shanghai Municipal Education Committee Key Laboratory of Molecular Imaging Probes and Sensors, Department of Chemistry, Shanghai Normal University, 100 Guilin Road, Shanghai 200234, People’s Republic of China; § Department of Chemistry, University of Bath, Bath, BA2 7AY, U.K.; ∥ School of Physics, Henan Normal University, Xinxiang 453007, People’s Republic of China; ⊥ Department of Chemistry, The University of Texas at Austin, 105 E 24th Street A5300, Austin, Texas 78712-1224, United States; # Key Laboratory for Advanced Materials and Joint International Research Laboratory of Precision Chemistry and Molecular Engineering, Feringa Nobel Prize Scientist Joint Research Center, School of Chemistry and Molecular Engineering, East China University of Science and Technology, 130 Meilong Road, Shanghai 200237, People’s Republic of China; ○ National Center for Drug Screening, State Key Laboratory of Drug Research, Shanghai Institute of Materia Medica, Chinese Academy of Sciences, 189 Guo Shoujing Road, Shanghai 201203, People’s Republic of China

## Abstract

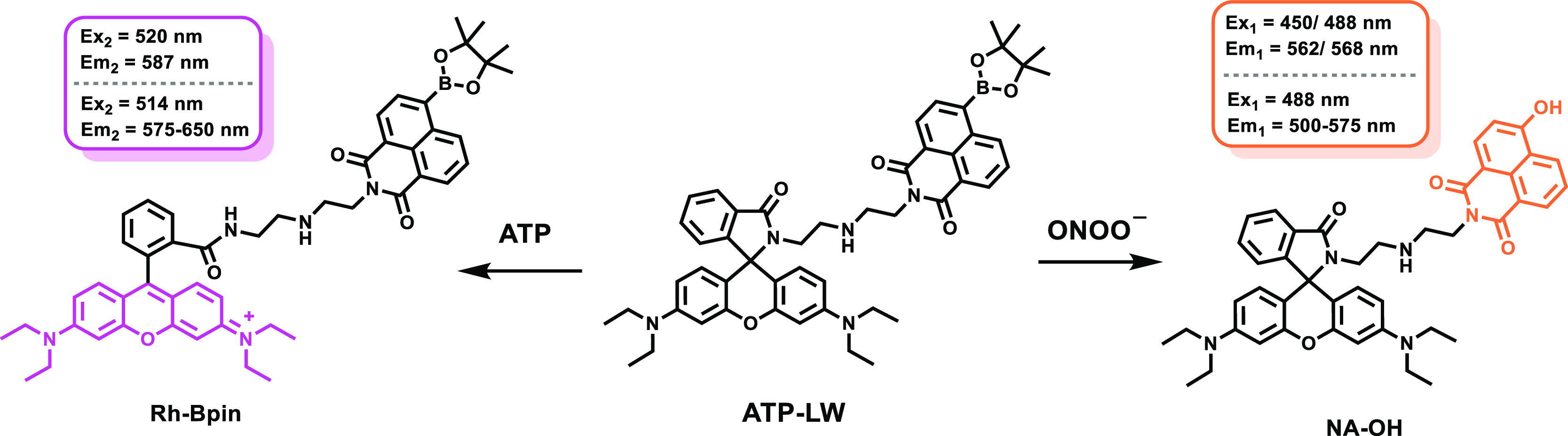

Changes in adenosine triphosphate (ATP) and peroxynitrite (ONOO^–^) concentrations have been correlated in a number of diseases including ischemia-reperfusion injury and drug-induced liver injury. Herein, we report the development of a fluorescent probe ATP-LW, which enables the simultaneous detection of ONOO^–^ and ATP. ONOO^–^ selectively oxidizes the boronate pinacol ester of ATP-LW to afford the fluorescent 4-hydroxy-1,8-naphthalimide product NA-OH (λ_ex_ = 450 nm, λ_em_ = 562 nm or λ_ex_ = 488 nm, λ_em_ = 568 nm). In contrast, the binding of ATP to ATP-LW induces the spirolactam ring opening of rhodamine to afford a highly emissive product (λ_ex_ = 520 nm, λ_em_ = 587 nm). Due to the differences in emission between the ONOO^–^ and ATP products, ATP-LW allows ONOO^–^ levels to be monitored in the green channel (λ_ex_ = 488 nm, λ_em_ = 500–575 nm) and ATP concentrations in the red channel (λ_ex_ = 514 nm, λ_em_ = 575–650 nm). The use of ATP-LW as a combined ONOO^–^ and ATP probe was demonstrated using hepatocytes (HL-7702 cells) in cellular imaging experiments. Treatment of HL-7702 cells with oligomycin A (an inhibitor of ATP synthase) resulted in a reduction of signal intensity in the red channel and an increase in that of the green channel as expected for a reduction in ATP concentrations. Similar fluorescence changes were seen in the presence of SIN-1 (an exogenous ONOO^–^ donor).

## Introduction

Adenosine-5′-triphosphate (ATP) has been referred to as the “molecular currency”.^1,2^ ATP concentrations range between 1 and 10 mM, with a 1000:1 ratio between ATP and adenosine diphosphate (ADP) typically prevailing.^3^ ATP aids the regulation of important cellular functions, including cellular movement,^4^ neurotransmission,^5^ and ion channel function.^6^ Disruption to ATP homeostasis is linked to a number of diseases, including ischemia, Parkinson’s disease, and hypoglycemia.^7^ The cause of this disruption is often ascribed to oxidative stress, which involves the production of highly reactive oxygen species (ROS) and reactive nitrogen species (RNS).^8,9^ In particular, peroxynitrite (ONOO^–^)^10^ is an RNS that is known to inhibit ATP production by oxidatively deactivating mitochondrial ATP synthase.^11^ Correlations between ATP and ONOO^–^ concentrations have been observed in a number of pathological events.^12−14^ Therefore, the development of a chemical tool that allows the real-time monitoring of these species simultaneously *in vitro* and *in vivo* would be highly desirable.

Fluorescence imaging has emerged as an attractive technology for the real-time and noninvasive detection of biomarkers in cellular and animal-based applications. Previously, several single-analyte fluorescent probes have been reported for the selective imaging of ONOO^–^.^15−17^ Moreover, fluorescent probes for the selective detection and visualization of ATP have been developed.^18^ However, to our knowledge no fluorescent probes capable of the simultaneous and independent imaging of ONOO^–^ and ATP have been reported. If available, such systems would allow the presumed close relationship between these two critical species to be monitored in real time. Herein, we report the construction of ATP-LW, a single fluorescent probe that enables the simultaneous detection of ONOO^–^ and ATP. Initial solution data established excellent water solubility, sensitivity, and high selectivity for ONOO^–^ and ATP using their respective detection emission profiles. As a preliminary proof-of-concept study for cellular applications, changes in ATP and ONOO^–^ associated with acetaminophen (APAP) were evaluated. This model was chosen due to the importance of preclinical tools for drug development in the screening of drug-induced liver injury (DILI) associated with drugs like APAP.^19−21^ Furthermore, this model was selected because APAP can result in a depletion of ATP and an increase in the levels of ONOO^–^ (Scheme S1).^22,23^ We believe ATP-LW could prove popular as a fluorescent tool in fundamental and clinical research. For example, the Sessler group has been developing type-II immunogenic cell death (ICD) inducers, where the ICD agent results in ROS-mediated ER stress and subsequent ATP release. We anticipate that ATP-LW will facilitate the rapid identification of type-II ICD inducers.^24^ It is important to note that we first disclosed our probe ATP-LW as a ChemRxiv preprint;^25^ however, we felt that the associated journal contribution (i.e., this article) would be substantially improved with the addition of biological experiments. Unfortunately, the Covid-19 pandemic severely hindered our research progress, and in the time between the ChemRxiv preprint and the present submission, the group of Tian et al. reported a somewhat similar structure for the simultaneous detection of ATP and H_2_O_2_.^26^ Pointedly, Merck recognized the potential of ATP-LW and has made it commercially available.^27^ However, details regarding the scope, utility, and even mode of use have yet to appear in a peer-reviewed forum. This report is designed to address this deficiency and to highlight the utility of what is now a commercially available fluorescent ONOO^–^ and ATP probe, ATP-LW.

At this point it is worth discussing the selectivity of boronate ester-based probes toward ONOO^–^ and H_2_O_2_. Since the seminal report by Sikora et al., numerous research groups, including our own, have determined that boronate ester-based fluorescent probes have a preferential reactivity toward ONOO^–^.^28−30^ As such, it is now universally accepted that ONOO^–^ reacts several orders of magnitude faster than hydrogen peroxide (microseconds vs hours).^31^ In addition an observed lack of response toward ONOO^–^ may indicate degradation of the fluorescent reporter by the highly reactive ONOO^–^.^20^

In recent years, multianalyte fluorescent probes have garnered attention owing to their enhanced precision for the study in question.^32,33^ These systems overcome the problems of using several independent fluorescent probes when seeking to understand the relationship between more than one biological species.^31^ Efforts from our group and others have led to the development of multianalyte fluorescent probes.^31^ For example, we reported AND-logic gate-like dual-analyte fluorescence scaffolds that permit the detection of ONOO^–^ and a second analyte.^34−38^ However, we believe that systems able to detect more than one analyte independently using different emission channels will prove particularly advantageous;^20^ this is because, in principle, dual-channel emission should enable the concurrent evaluation of each individual species, whereas AND-logic systems only provide information on the synergy of the two species.

**Scheme 1:**
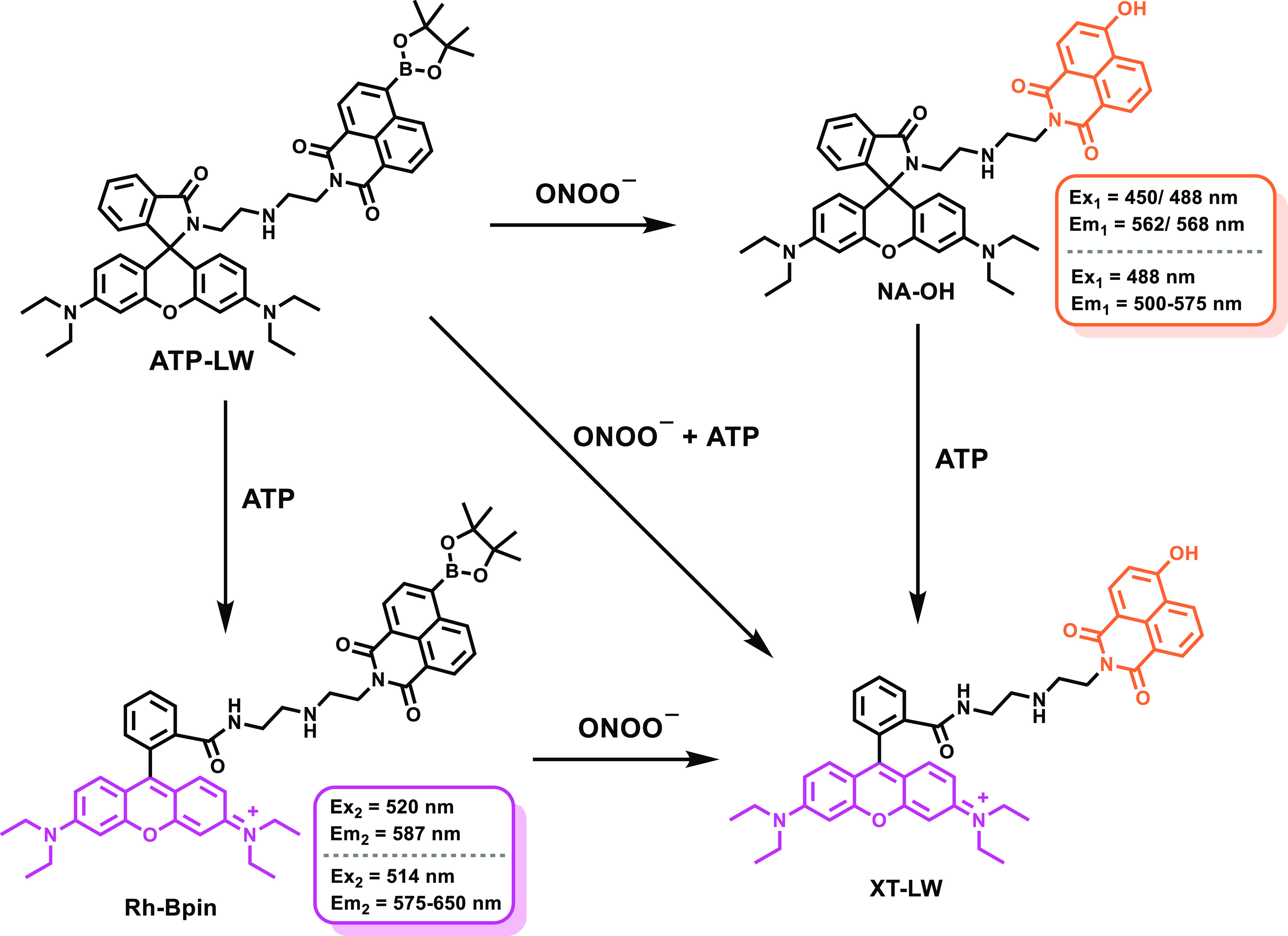
Chemical Structure of ATP-LW and Its Fluorescence “Turn On” Mechanism in the Presence of ONOO^–^, ATP, and ONOO^–^/ATP) The addition of ONOO^–^ leads to formation of compound NA-OH, with a maximum emission at 562 nm when excited at 450 nm and a maximum at 568 nm when excited at 488 nm in PBS buffer solution (10 mM, v/v, EtOH/H_2_O = 1/99, pH = 7.40). The presence of ATP affords the product Rh-Bpin, with a maximum emission at 587 nm when excited at 520 nm in PBS buffer solution (10 mM, v/v, EtOH/H_2_O = 1/99, pH = 7.40).

In this work, we have used a rhodamine lactam/1,8-naphthalimide hybrid structure as a scaffold to create the dual-analyte fluorescent probe ATP-LW (Scheme 1).^39^ ATP-LW was synthesized over three steps (Scheme S2). The first step of the synthesis involves a Miyaura borylation reaction, which forms intermediate NA-Bpin. Diethylenetriamine was then added to a solution of rhodamine B in methanol. This reaction afforded intermediate Rh-AM as a light orange solid. Condensation between NA-Bpin and Rh-AM in ethanol then afforded ATP-LW. The chemical structure of ATP-LW was fully characterized using ^1^H NMR and ^13^C NMR spectroscopy, as well as high-resolution mass spectrometry.

## Results and Discussion

**Figure 1:**
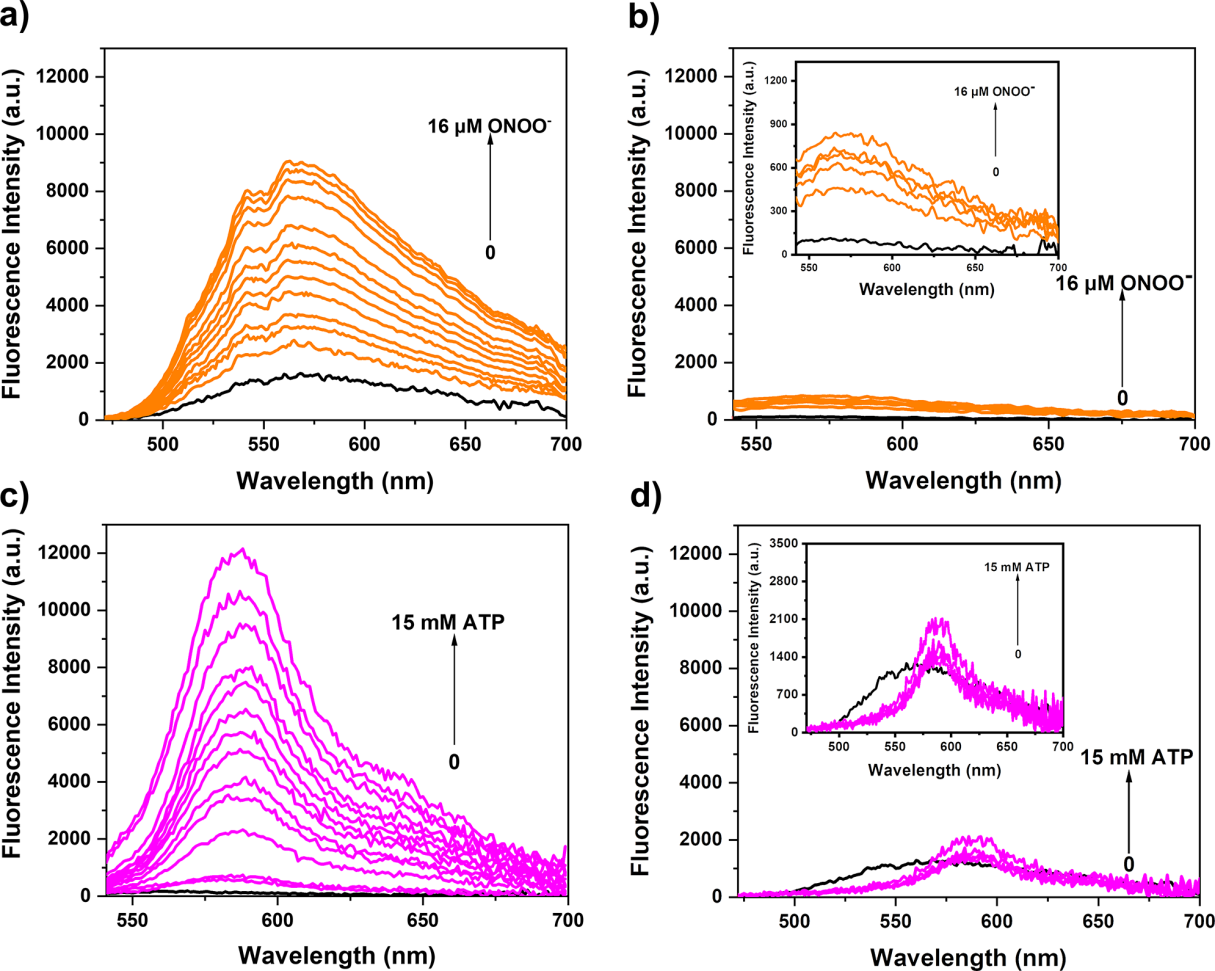
Changes in the fluorescence emission intensity of ATP-LW (15 μM) seen upon the addition of ONOO^–^ (from 0 to 16 μM) in modified PBS buffer (10 mM, v/v, EtOH/H_2_O = 1/99, pH = 7.40) after 1 min. (a) Sample excited at 450 (bandwidth 8) nm; (b) sample excited at 520 (bandwidth 8) nm. Inset: Enlarged views of the spectra in (b). Changes in the fluorescence emission intensity of ATP-LW (15 μM) seen upon the addition of ATP (from 0 to 15 mM) in modified PBS buffer solution (10 mM, v/v, EtOH/H_2_O = 1/99, pH = 7.40) after 100 min. (c) Sample excited at 520 (bandwidth 8) nm; (d) sample excited at 450 (bandwidth 8) nm. Inset: Enlarged views of the spectra in (d).

In initial studies, the changes in the UV–vis absorption spectral features of ATP-LW in the presence of ONOO^–^ and ATP were investigated in aqueous media (PBS buffer, 10 mM, v/v, EtOH/H_2_O = 1/99, pH = 7.40). The addition of ONOO^–^ produced a new absorption peak at 445 nm, which is consistent with the known intramolecular charge transfer (ICT) process seen in 4-hydroxy-1,8-naphthalimide products (Figure S1).^40^ Similarly, the addition of ATP to ATP-LW resulted in an increase in the absorption intensity at 553 nm. This was taken as evidence for the ATP-induced opening of the spirolactam ring on the rhodamine fluorophore (Figure S2).^18^ ATP-LW was initially nonfluorescent, presumably as the result of quenching by the boronic ester on the 1,8-naphthalimide unit, and the ring-closed form of rhodamine being inherently nonfluorescent. Upon the addition of ONOO^–^ (0–16 μM), an increase in the fluorescence intensity at 562 nm/568 nm was observed upon excitation at 450/488 nm (Figure 1a, Figures S7–S10). A minimal fluorescence increase was observed for light excitation at 520 nm upon the addition of ONOO^–^ (0–16 μM) (Figure 1b). The formation of a 4-hydroxy-1,8-naphthalimide product upon treatment of ATP-LW with ONOO^–^ was confirmed by LC-MS analysis (Scheme S3, Figures S24 and S25). A 488 nm excitation wavelength was used for the measurements in aqueous solution since it was the excitation wavelength for cellular imaging experiments (green channel, λ_em_ = 500–575 nm, λ_ex_ = 488 nm).

Subsequently we evaluated the ability of ATP-LW to detect ATP. A large increase in fluorescence intensity at 587 nm (>80-fold, see Figure 1c and Figure S4) was observed following the addition of ATP (0–15 mM) to an aqueous solution of ATP-LW when the samples were excited at 520 nm. However, the addition of ATP (0–15 mM) to ATP-LW resulted in only a small increase in the fluorescence intensity upon excitation at either 450 (Figure 1d) or 488 nm (Figure S6). The disparity in emission and excitation profiles seen for ATP and ONOO^–^ provides support for the notion that ATP-LW may be used to detect separately, albeit contemporaneously, these two important analytes.

We then explored the fluorescence response of ATP-LW in the presence of both ONOO^–^ and ATP. It was found that the peak at 589 nm for ATP-LW retained sensitivity to changes in the ATP concentration even after the addition of ONOO^–^ (16 μM) when excitation was effected at 520 nm (Figure S13). Conversely, the emission peaks at 562 nm (Figure S14) or 569 nm (Figure S15) were seen to change as a function of the ONOO^–^ concentration, but were only slightly influenced by the presence or absence of ATP (15 mM). These results were taken as a further indication that ATP-LW may be used to detect ONOO^–^ and ATP independently, on the condition that two different excitation wavelengths are used (Scheme 1). We appreciate that the two emission peaks for ATP-LW are not well separated. As such, it is difficult to ascertain whether Förster resonance energy transfer (FRET) is occurring between the naphthalimide and rhodamine that make up ATP-LW.^39^ However, on the basis of density functional theoretical analysis we believe that FRET does not occur to a significant extent between the two fluorophores present in ATP-LW (Figure S22).

**Figure 2:**
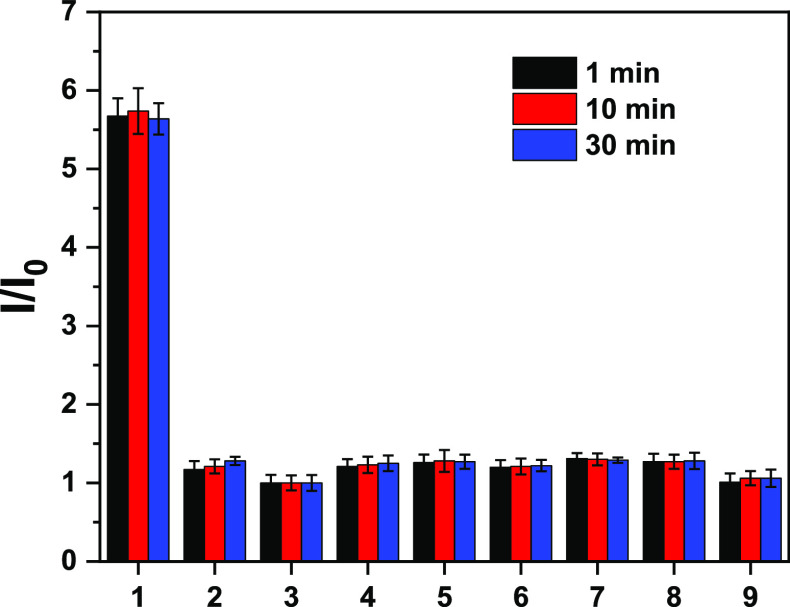
Selectivity bar chart for probe ATP-LW (15 μM) in PBS buffer solution (10 mM, v/v, EtOH/H_2_O = 1/99, pH = 7.40) with ONOO^–^ (16 μM) or other ROS. (1) ONOO^–^; (2) H_2_O_2_ (100 μM); (3) probe ATP-LW alone; (4) HOCl (100 μM); (5) ROO^•^ (200 μM); (6) ^•^OH (100 μM); (7) O_2_^•–^ (100 μM); (8) ^1^O_2_ (100 μM); (9) APAP (20 mM); λ_ex/em_= 450 (bandwidth 8) nm/562 nm. Time points were taken at 1 min (black bars), 10 min (red bars), and 30 min (blue bars).

**Figure 3:**
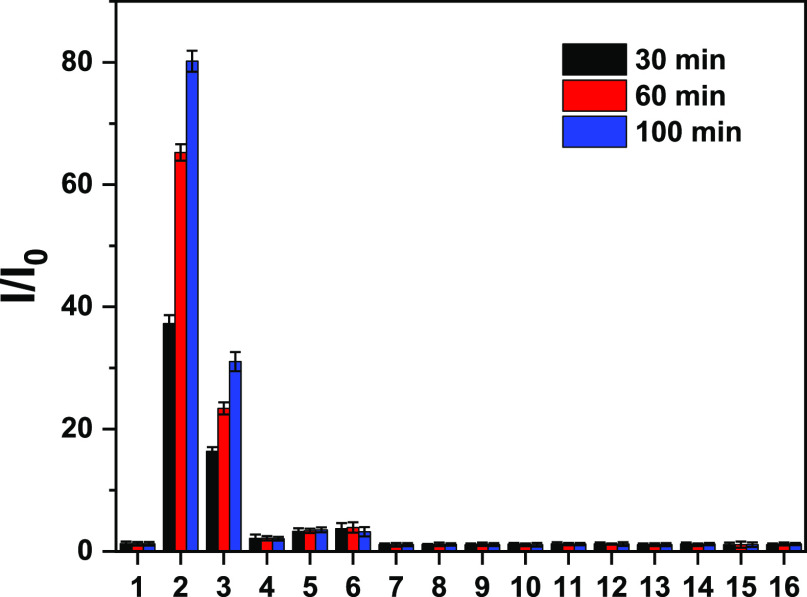
Selectivity bar chart of ATP-LW (15 μM) in PBS buffer solution (10 mM, v/v, EtOH/H_2_O = 1/99, pH = 7.40) upon treatment with ATP (15 mM) or other potential interferents. (1) Probe ATP-LW alone; (2) ATP (15 mM); (3) adenosine diphosphate (ADP, 10 mM); (4) uridine 5′-triphosphate, trisodium salt (UTP trisodium salt 10 mM); (5) guanosine 5′-triphosphate, disodium salt (GTP disodium salt, 10 mM); (6) cytidine 5′-triphosphate disodium salt (CTP disodium salt, 10 mM); (7) cysteine (1 mM); (8) glutathione (1 mM); (9) homocysteine (1 mM); (10) KCl (6 mM); (11) CaCl_2_ (2 mM); (12) MgCl_2_ (1 mM); (13) CuCl_2_ (100 μM); (14) ZnCl_2_ (100 μM); (15) NaCl (100 mM); (16) APAP (20 mM); λ_ex/em_ = 520 (bandwidth 8) nm/587 nm. Time points were taken at 30 min (black bars), 60 min (red bars), and 100 min (blue bars).

We then evaluated the selectivity of the probe ATP-LW toward a variety of potential biologically relevant interferents (Figures 2 and 3). Other ROS, such as H_2_O_2_ and HOCl, led to no change in the fluorescence intensity of ATP-LW under conditions identical to those used to study ONOO^–^ and ATP (Figure 2). However, exposure of ATP-LW to ADP (10 mM) led to an enhancement in the fluorescence intensity (Figure 3). Since the concentration of ATP is around 1000-fold higher than ADP in cells,^3^ as noted above, it seems highly unlikely that this cross reactivity will preclude the use of ATP-LW for the effective detection of ATP in cells.

Exposure to ONOO^–^ resulted in a statistically significant fluorescence increase that was instantaneous on the laboratory time scale (Figure S16), while the reaction of ATP-LW and ATP required approximately 100 min to reach saturation (Figure S17). We next confirmed that ATP-LW exhibits good stability over a pH range from 3 to 11 (Figures S11 and S12). The fluorescence emission of a test solution formed by treating ATP-LW with ONOO^–^ was found to decrease at lower pH, a finding that could reflect the known decomposition of ONOO^–^ in acidic media (Figure S11).^41^ The fluorescence intensity at 587 nm was also found to decrease at higher pH (pH > 8), a result ascribed to the hydrolysis of ATP under basic conditions (Figure S12).^42^

**Figure 4:**
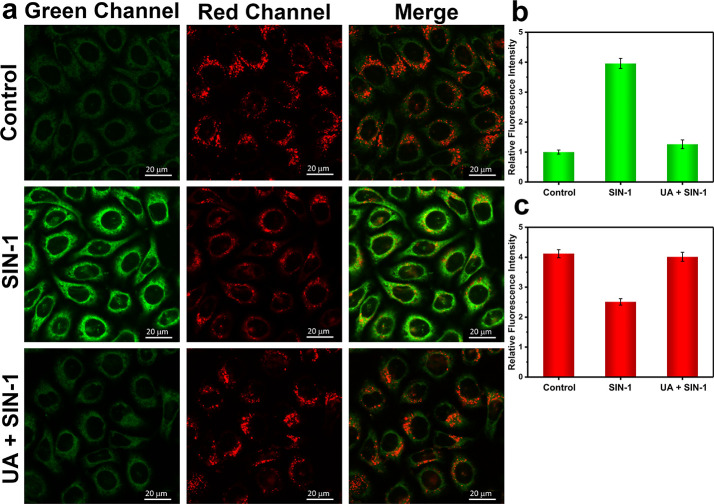
One-photon confocal imaging of ONOO^–^ and ATP levels in hepatocytes treated with SIN-1 or SIN-1/uric acid. (a) One-photon fluorescence images of HL-7702 cells recorded after the addition of SIN-1 (1 mM, 1 h) and uric acid (500 μM, 1 h) with monitoring over the green (ONOO^–^) and red (ATP) channels. Control group: Cells were stained with probe ATP-LW (20 μM) for 20 min. SIN-1 group: Cells were incubated with SIN-1 (1 mM) for 1 h, then stained with probe ATP-LW (20 μM) for 20 min. UA + SIN-1 group: Cells were pretreated with uric acid (500 μM) for 1 h and followed by adding SIN-1 (1 mM) for 1 h and then stained with probe ATP-LW (20 μM) for 20 min. Green fluorescence channel for ONOO^–^: λ_ex_ = 488 nm, λ_em_ = 500–575 nm. Red fluorescence channel for ATP: λ_ex_ = 514 nm, λ_em_ = 575–650 nm. (b) Green relative fluorescence intensity output of three groups. (c) Red relative fluorescence intensity output of three groups. Note: The green fluorescence intensity of the control group is defined as 1.0. The data are expressed as the mean ± SD. Concordant results were obtained from five independent experiments.

The above results prompted us to explore the use of ATP-LW for imaging live cells. First, we confirmed using an MTT assay that ATP-LW was non-toxic to HL-7702 cells over concentrations ranging from 0 to 1 mM with an incubation time of 24 h (Figure S27). The ability of ATP-LW to image ONOO^–^ and ATP in living cells was then evaluated using excitation wavelengths of 488 and 514 nm, respectively. ATP-LW demonstrated a clear “turn on” response upon the addition of 3-morpholinosydnonimine hydrochloride (SIN-1, a donor of ONOO^–^)^43^ when monitored using the green channel (Figure 4a and b). Meanwhile, a 0.61-fold decrease in the red channel was observed as compared to the control group (Figure 4c). We then evaluated crosstalk between the two channels, and as expected, no appreciable fluorescence was observed in either channel 3 (λ_ex_ = 488 nm, λ_em_ = 575–650 nm) or channel 4 (λ_ex_ = 514 nm, λ_em_ = 520–575 nm) when HL-7702 cells were treated with ATP-LW and simultaneously exposed to ATP and SIN-1 (Figure S28). Having confirmed the absence of crosstalk, fluorescence changes using the ONOO^–^ scavenger uric acid were evaluated.^44^ The addition of uric acid (500 μM) and SIN-1 (1.0 mM) led to a 0.32-fold decrease in the average green fluorescence intensity and 1.60-fold enhancement in the average red fluorescence intensity, when compared with the corresponding SIN-1 group (as determined by monitoring the green and red channels, respectively, Figure 4). These results are consistent with suggestions in the literature that an increase in the ONOO^–^ concentration can result in depletion of ATP.^22^

**Figure 5:**
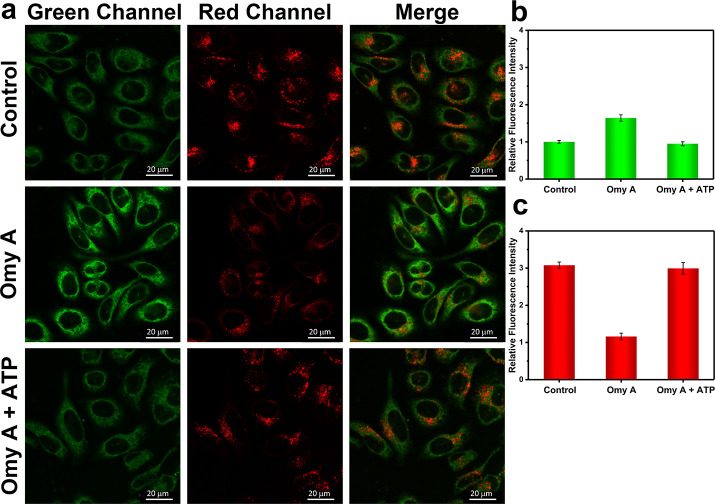
One-photon confocal imaging of ONOO^–^ and ATP levels in hepatocytes treated with omy A or omy A/ATP. (a) One-photon fluorescence images of HL-7702 cells with the addition of omy A (25 μM, 1 h) and ATP (10 mM, 1 h) for green (ONOO^–^) and red (ATP) channels. Control group: Cells were stained with probe ATP-LW (20 μM) for 20 min. Omy A group: Cells were incubated with omy A (25 μM) for 1 h, then stained with probe ATP-LW (20 μM) for 20 min. Omy A + ATP group: Cells were pretreated with omy A (25 μM) for 1 h followed by adding ATP (10 mM) for 1 h and then stained with probe ATP-LW (20 μM) for 20 min. Green fluorescence channel for ONOO^–^: λ_ex_ = 488 nm, λ_em_ = 500–575 nm. Red fluorescence channel for ATP: λ_ex_ = 514 nm, λ_em_ = 575–650 nm. (b) Green relative fluorescence intensity output of three groups. (c) Red relative fluorescence intensity output of three groups. Note: The green fluorescence intensity of the control group is defined as 1.0. The data are expressed as the mean ± SD. Concordant results were obtained from five independent experiments.

We then set out to evaluate how imbalances in the energy metabolism instigated by obstructing ATP production can influence production of ONOO^–^. Oligomycin A (omy A) inhibits ATP synthase by blocking its F_O_ unit.^45^ As shown in Figure 5, after hepatocytes were incubated with omy A (25 μM) for 1 h, a 38% decrease in the red channel signal relative to the initial level was seen (Figure 5c). Concurrently, a 1.64-fold increase in the green channel intensity was seen, as expected for an increase in the ONOO^–^ levels as compared to healthy hepatocytes (Figure 5b). The addition of exogenous ATP (10 mM) yielded a green channel intensity that was 95% of the control group (Figure 5b), while the intensity of the red channel increased to almost the same level as the control group (Figure 5c). While not a proof, these findings support the conclusion that the addition of exogenous ATP induces recovery^26^ and that ROS/RNS production is affected by mitochondrial damage to ATP synthesis, leading to an increase of ONOO^–^. These results are not surprising since it is known that omy A induces cellular apoptosis via ATP inhibition;^46,47^ moreover, oxidative stress (i.e., ONOO^–^) is an associated factor in apoptosis-related cell death.^48^ As such, our results support the conclusion that omy A induces the production of ONOO^–^ in a cellular environment via ATP inhibition.

**Figure 6:**
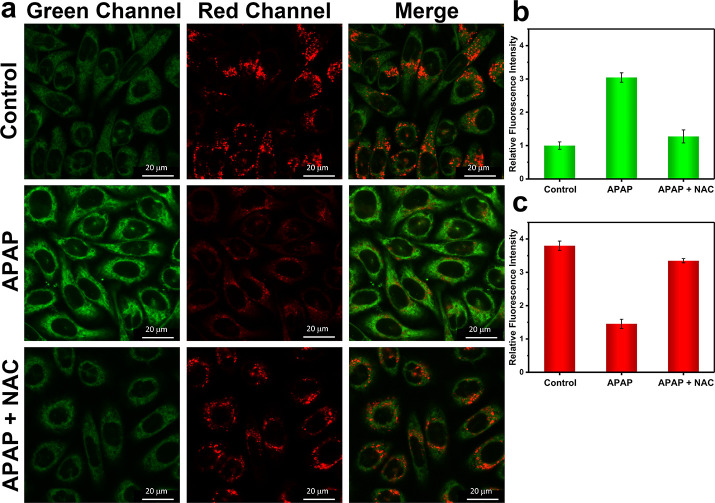
One-photon confocal images of APAP-induced injury and its remediation by NAC in HL-7702 cells. (a) One-photon fluorescence images of HL-7702 cells with the addition of APAP (15 mM, 2 h) and NAC (2 mM, 2 h) for green (ONOO^–^) and red (ATP) channels. Control group: Cells were stained with probe ATP-LW (20 μM) for 20 min. APAP group: Cells were incubated with APAP (15 mM) for 2 h and then stained with probe ATP-LW (20 μM) for 20 min. APAP + NAC group: Cells were pretreated with NAC (2 mM) for 2 h and then incubated with APAP (15 mM) for 2 h, followed by staining with probe ATP-LW (20 μM) for another 20 min. Green fluorescence channel for ONOO^–^: λ_ex_ = 488 nm, λ_em_ = 500–575 nm. Red fluorescence channel for ATP: λ_ex_ = 514 nm, λ_em_ = 575–650 nm. (b) Green relative fluorescence intensity output of three groups. (c) Red relative fluorescence intensity output of three groups. Note: The green fluorescence intensity of the control group is defined as 1.0. The data are expressed as the mean ± SD. Concordant results were obtained from five independent experiments.

ROS and RNS are regarded as biomarkers in DILI and are thus analytes that have been frequently targeted using fluorescent probes.^19,21^ Previous studies confirm that ATP production is decreased by exposure to APAP.^22,49,50^ APAP-induced hepatoxicity was thus chosen as the model to investigate whether ATP-LW could detect ONOO^–^ and ATP, since these two species could serve as early diagnostic biomarkers. Treatment of HL-7702 cells with APAP produced a marked increase in the fluorescence of the green channel and a significant decrease in the fluorescence in the red channel (Figure 6). This finding underscores how upregulation of intracellular ONOO^–^ and depletion of ATP occur after administration of APAP while serving to illustrate the ability of our probe to detect concentration changes of these two biomarkers via one-photon confocal imaging in a DILI cellular model. This was further confirmed using *N*-acetyl-l-cysteine (NAC), which is a precursor for the substrate (l-cysteine) in the synthesis of reduced glutathione (GSH) and commonly used for the treatment of APAP overdose.^51,52^ GSH is capable of eliminating ONOO^–^, and as such has been used to help treat APAP overdoses.^53,54^ Upon addition of NAC, the fluorescence intensity in the green channel decreased and that of the red channel increased (Figure 6). This finding is thus consistent with the reduction of ONOO^–^ and an increase in the ATP concentration under these conditions.

**Figure 7:**
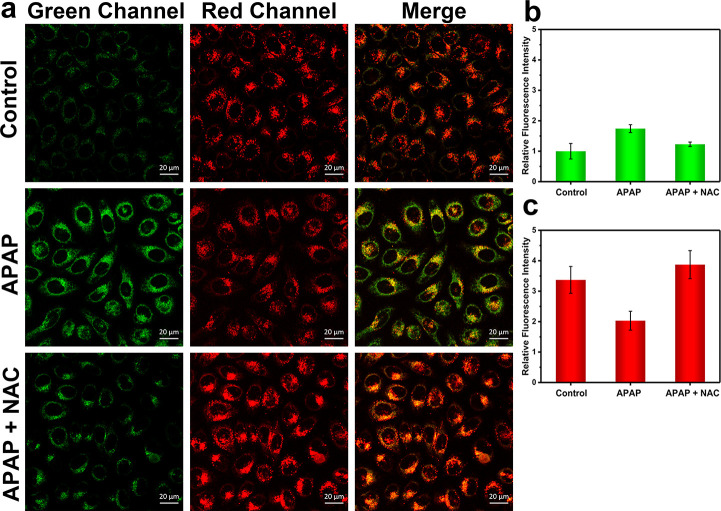
Two-photon fluorescence images of APAP-induced injury with HL-7702 cells. (a) Two-photon fluorescence images of HL-7702 cells with the addition of APAP (20 mM, 2 h) and NAC (2 mM, 2 h) for green (ONOO^–^) and red (ATP) channels. Control group: Cells were stained with probe ATP-LW (20 μM) for 20 min. APAP group: Cells were incubated with APAP (20 mM) for 2 h and then stained with probe ATP-LW (20 μM) for 20 min. APAP + NAC group: Cells were pretreated with NAC (2 mM) for 2 h and then incubated with APAP (20 mM) for 2 h, followed by staining with probe ATP-LW (20 μM) for another 20 min. Two-photon green fluorescence channel for ONOO^–^: λ_ex_ = 976 nm, λ_em_ = 500–575 nm. Two-photon red fluorescence channel for ATP: λ_ex_ = 1028 nm, λ_em_ = 575–680 nm. (b) Green relative fluorescence intensity output of three groups. (c) Red relative fluorescence intensity output of three groups. Note: The green fluorescence intensity of the control group is defined as 1.0. The data are expressed as the mean ± SD. Concordant results were obtained from five independent experiments.

Our attention then turned to two-photon imaging^55^ of ONOO^–^ and ATP using ATP-LW, with the same cell models used above. The results (Figure 7) served to confirm the ability of ATP-LW to image both ONOO^–^ and ATP, using an excitation of 976 nm for the former and 1028 nm for the latter, and we extended the emission range for the red channel (i.e., 575–680 nm).

## Conclusion

In summary, we have developed a novel dual-analyte fluorescent probe (ATP-LW), which provides a fluorescence response toward ONOO^–^ and ATP simultaneously by means of different excitation wavelengths. Probe ATP-LW comprises two responsive units that are expected to react independently with ONOO^–^ and ATP, respectively.^31^ Upon the addition of ONOO^–^ alone, the 4-hydroxy-1,8-naphthalimide subunit luminesces (λ_ex_ = 450 nm, λ_em_ = 562 nm or λ_ex_ = 488 nm, λ_em_ = 568 nm); conversely, when ATP alone is present, the rhodamine ring opens and luminesces (λ_ex_ = 520 nm, λ_em_ = 587 nm). In order to detect ONOO^–^ and ATP in cellular milieus with minimal crosstalk, we choose to monitor the emission over the green channel (λ_ex_ = 488 nm, λ_em_ = 500–575 nm) and red channel (λ_ex_ = 514 nm, λ_em_ = 575–650 nm). It was found that by using ATP-LW and two different channels it is possible to monitor concurrently the enhancement of ONOO^–^ and depletion of ATP during APAP-induced hepatotoxicity. This monitoring provides support for the proposed signaling pathways for APAP-induced toxicity wherein ONOO^–^ increases and ATP depletion are thought to be responsible for hepatic necrosis.^56^ We anticipate that ATP-LW can be extended to image fluctuations of these two biomarkers in other diseases, such as ischemia-reperfusion injury.^14^

## Supporting Information Available

Materials and instruments used; mechanism of APAP-induced toxicity; synthesis and analysis of ATP-LW, including mass spectrometric analyses and NMR spectra; methods used for the generation of ROS/RNS; UV–vis and fluorescence analyses; theoretical calculations and transient absorption spectra; cell culture protocols; MTT assay and fluorescence imaging of live cells (PDF)
