# Supplementary material for: Dual-Channel Fluorescent Probe for the Simultaneous Monitoring of Peroxynitrite and Adenosine-5′-triphosphate in Cellular Applications
Source: J Am Chem Soc. 2021 Dec 21;144(1):174–83. doi: 10.1021/jacs.1c07954 (PMC8759067; doi:10.1021/jacs.1c07954)
Supplement: Supplementary file 1 — ja1c07954_si_001.pdf [file ja1c07954_si_001.pdf]

Dual-channel fluorescent probe for the simultaneous monitoring of peroxynitrite and adenosine-5'-triphosphate in cellular applications

Luling Wu,[abc‡] Jihong Liu,[a‡] Xue Tian,[c‡] Robin R. Groleau,[c] Beidou Feng,[d] Yonggang Yang,[h] Adam C. Sedgwick,[e] Hai-Hao Han,[f*] Yang Wang,[b] Han-Min Wang,[g] Fang Huang,[a] Steven D. Bull,[c] Hua Zhang,[d*] Chusen Huang[b*] Yi Zang,[g] Jia Li,[g] Xiao-Peng He,[f] Ping Li,[a*] Bo Tang,[a] Tony D. James,[acd*] and Jonathan L. Sessler[e*]

a. College of Chemistry, Chemical Engineering and Materials Science, Key Laboratory of Molecular and Nano Probes, Ministry of Education, Collaborative Innovation Center of Functionalized Probes for Chemical Imaging in Universities of Shandong, Institutes of Biomedical Sciences, Shandong Normal University, Jinan 250014, People's Republic of China. Email: lip@sdnu.edu.cn

b. The Education Ministry Key Laboratory of Resource Chemistry, Shanghai Key Laboratory of Rare Earth Functional Materials, and Shanghai Municipal Education Committee Key Laboratory of Molecular Imaging Probes and Sensors, Department of Chemistry, Shanghai Normal University, 100 Guilin Road, Shanghai 200234, China. Email: huangcs@shnu.edu.cn

c. Department of Chemistry, University of Bath, Bath, BA2 7AY, UK. Email: t.d.james@bath.ac.uk

d. School of Chemistry and Chemical Engineering, Henan Normal University, Xinxiang 453007, P. R. China. Email: zhanghua1106@163.com

e. Department of Chemistry, The University of Texas at Austin, 105 E 24th street A5300, Austin, TX 78712-1224, USA. Email: sessler@cm.utexas.edu

f. Key Laboratory for Advanced Materials and Joint International Research Laboratory of Precision Chemistry and Molecular Engineering, Feringa Nobel Prize Scientist Joint Research Center, School of Chemistry and Molecular Engineering, East China University of Science and Technology, 130 Meilong Rd., Shanghai 200237, China. Email: hanhaihao@126.com

g. National Center for Drug Screening, State Key Laboratory of Drug Research, Shanghai Institute of Materia Medica, Chinese Academy of Sciences, 189 Guo Shoujing Rd., Shanghai 201203, P. R. China.

h. School of Physics, Henan Normal University, Xinxiang 453007, P. R. China.

‡These authors contribute equally.

Table of contents

1. **Materials and instruments**
2. **Mechanism of APAP-induced toxicity**
3. **Synthesis of probe ATP-LW**
4. **Generation of various ROS/RNS**
5. **UV-Vis and fluorescence analyses**
6. **Theoretical calculations to confirm absence of FRET**
7. **Transient absorption studies confirming the absence of electron transfer**
8. **Mass spectrometric analyses**
9. **Protocols for cell culture**
10. **MTT assay and fluorescence imaging in live cells**
11. **NMR spectra**
12. **Author contributions**
13. **References**

1. Materials and instruments

All starting materials, reagents and solvents were purchased from commercial sources and used without further purification. 3-Morpholinosydnonimine hydrochloride (SIN-1) was purchased from Sigma-Aldrich Co., LLC. Uric acid was purchased from Energy Chemical. 4-Acetamidophenol (APAP) and *N*-acetyl-L-cysteine (NAC) were purchased from Shanghai Aladdin Biochemical Technology Co., Ltd. Oligomycin A was purchased from MedChemExpress. Adenosine 5'-triphosphate (ATP) disodium salt hydrate purchased from Acros Organics was used to represent ATP for all solution experiments, and molecular weight is 551.14 (anhydrous basis) for the calculation of ATP concentrations. ATP disodium salt purchased from MedChemExpress for the exogenous addition of ATP in the cell work. All water was deionized which is generated by a reverse osmosis (Ultra-Purified Type I, 18.2 Megohm water). Thin-layer chromatography was performed using commercially available Fluorochem aluminum-backed plates coated with a layer of silica gel (60 Å) with fluorescent indicator UV254. These plates were visualized using ultraviolet light with a wavelength of either 254 or 365 nm. Silica gel column chromatography was carried out using Sigma Aldrich 60 Å silica gel (200-400 mesh). Nuclear Magnetic Resonance (NMR) spectroscopy experiments were performed in chloroform-*d* on either an Agilent ProPlus 500 spectrometer, or in the case of **ATP-LW** a 500 MHz Bruker Avance III with a prodigy cryoprobe. Proton decoupling was used for all ^{13}C NMR spectra. ^{1}H and ^{13}C NMR chemical shifts (δ) are quoted in parts per million (ppm) and are referenced to the deuterated solvent. Fluorescence measurements were performed on a BMG Labtech CLARIOstar® plate reader using Greiner Bio-One microplates (96-well, PS, f-bottom (chimney well), black-walled). Data were collected via the BMG Labtech Clariostar data analysis software package MARS. LC−MS analysis were performed using an Agilent QTOF 6545 with Jetstream ESI spray source coupled to an Agilent 1260 Infinity II Quat pump HPLC with 1260 autosampler, column oven compartment and variable wavelength detector (VWD). MTT assay was performed using a Triturus microplate reader. One-photon confocal imaging was performed on Leica SP8 high-resolution fluorescence microscope. The two-photon fluorescence images were taken using a LSM 880 NLO microscope (Zeiss Co., Ltd. Germany).

2. Mechanism of APAP-induced toxicity

CYP2E1 is implicated in the metabolism of APAP to generate *N*-acetyl-*p*-benzoquinone imine (NAPQI) which highly toxic to the liver.[1-2] It is believed that the toxicity of NAPQI is partly due to conjunction with crucial proteins and with the antioxidant glutathione (GSH) resulting in an increase in vulnerability towards oxidative stress.[3-4] NAPQI interferes with complex I/II of the mitochondrial electron transport chain, leading to a release of electrons. The electrons are captured by molecular oxygen, inducing the over-production of superoxide ($O_2^{\bullet-}$).[5-6] Subsequently, endogenous nitric oxide can react with $O_2^{\bullet-}$ to generate $ONOO^-$[7] which is a scavenger of GSH.[8] However, NAC is a precursor for the substrate (L-cysteine) in the synthesis of hepatic GSH and commonly used for the treatment of APAP overdose.[9] The mitochondrial permeability transition (MPT) is a vital event in apoptotic and necrotic cell death.[10-11] An increase of reactive oxygen species (ROS, e.g. H_2O_2)[12] or RNS (e.g. $ONOO^-$)[13] promotes MPT.[3] The onset of MPT increases mitochondrial membrane permeability and changes the mitochondrial electrochemical gradient (*i.e.* membrane potential and pH gradient),[14-15] resulting in reduced ATP production.[16-18] In addition, defective or impaired ATP production has been associated with interactions between NAPQI and ATP-synthase α-subunit.[19]

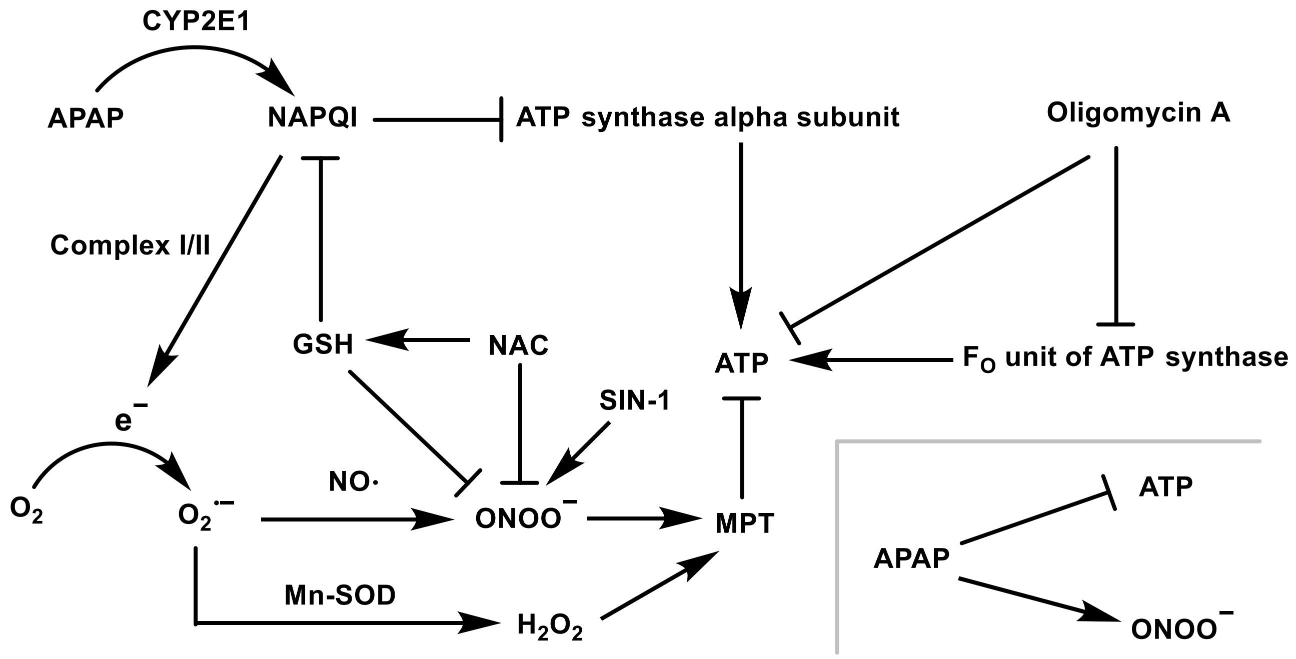

Scheme S1. Mechanism of APAP-induced toxicity. The depletion of ATP and increase of ONOO$^-$ cause hepatic necrosis.[3] The arrows indicate positive regulation, bars indicate negative regulation. Mn-SOD, manganese superoxide dismutase.

3. Synthesis of probe ATP-LW

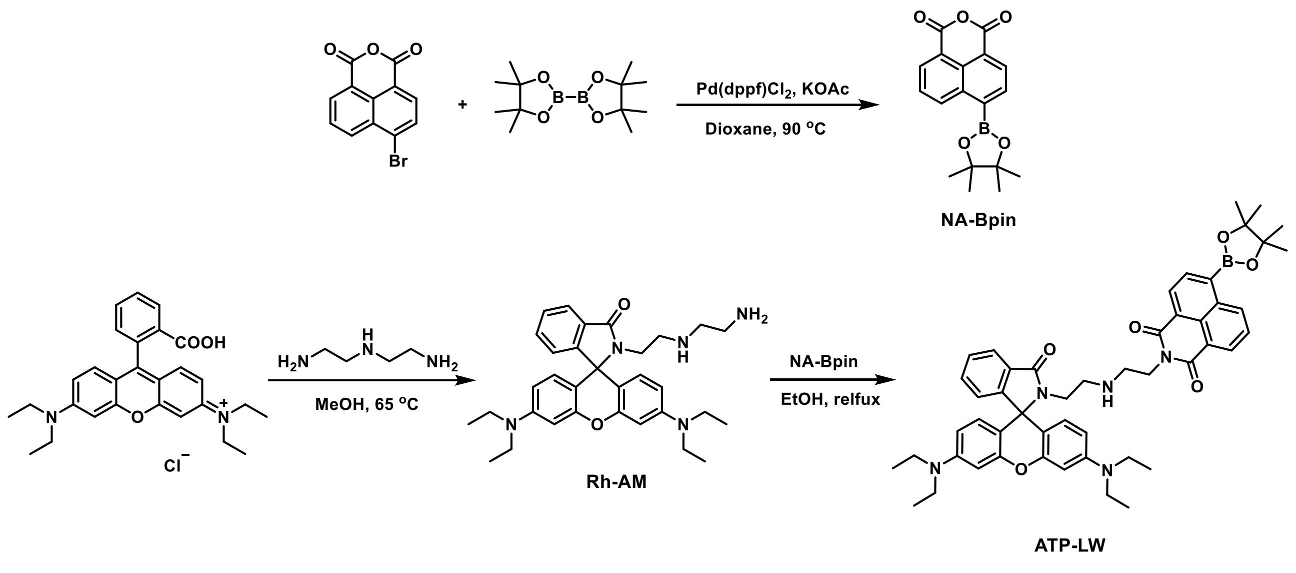

Scheme S2. Synthesis of probe **ATP-LW**.

Synthesis of NA-Bpin

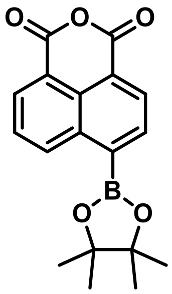

NA-Bpin

NA-Bpin was synthesized using revised published literature procedures.[20] Characterization data were consistent with previous literature reports.[20] ^{1}H NMR (500 MHz, $CDCl_3$) δ_H 9.22 (dd, J = 8.5, 1.1 Hz, 1H, Ar*H*), 8.61 (dd, J = 7.3, 1.0 Hz, 1H, Ar*H*), 8.57 (d, J = 7.2 Hz, 1H, Ar*H*), 8.34 (d, J = 7.3 Hz, 1H, Ar*H*), 7.83 (dd, J = 8.4, 7.3 Hz, 1H, Ar*H*), 1.47 (s, 12H, 4 x CH_3). ^{13}C NMR (126 MHz, $CDCl_3$) δ_C 160.9, 160.8, 136.6, 136.2, 135.5, 133.2, 132.0, 130.0, 127.7, 120.9, 118.8, 85.0, 25.1.

Synthesis of Rh-AM

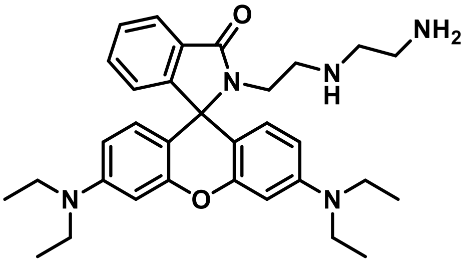

Rh-AM

Rh-AM was synthesized using revised reported literature procedures.[21] Characterization data were consistent with previous literature reports.[21] ^{1}H NMR (500 MHz, $CDCl_3$) δ_H 7.91 – 7.85 (m, 1H, Ar*H*), 7.42 (dd, J = 5.6, 3.1 Hz, 2H, Ar*H*), 7.10 – 7.05 (m, 1H, Ar*H*), 6.42 (d, J = 8.8 Hz, 2H, Ar*H*), 6.37 (d, J = 2.6 Hz, 2H, Ar*H*), 6.27 (d, J = 2.6 Hz, 1H, Ar*H*), 6.25 (d, J = 2.6 Hz, 1H, Ar*H*), 3.32 (q, J = 7.1 Hz, 8H, 4 x CH_3CH_2), 3.27 (t, J = 6.5 Hz, 2H, CH_2), 2.63 (t, J = 5.9 Hz, 2H, CH_2), 2.48 (t, J = 5.9 Hz, 2H, CH_2), 2.40 (t, J = 6.5 Hz, 2H, CH_2), 1.15 (t, J = 7.1 Hz, 12H, 4 x CH_3). ^{13}C NMR (126 MHz, $CDCl_3$) δ_C 168.7, 153.7, 153.4, 148.9, 132.5, 131.3, 128.9, 128.1, 123.9, 122.9, 108.2, 105.7, 97.9, 65.2, 51.5, 47.9, 44.5, 41.6, 40.3, 12.7.

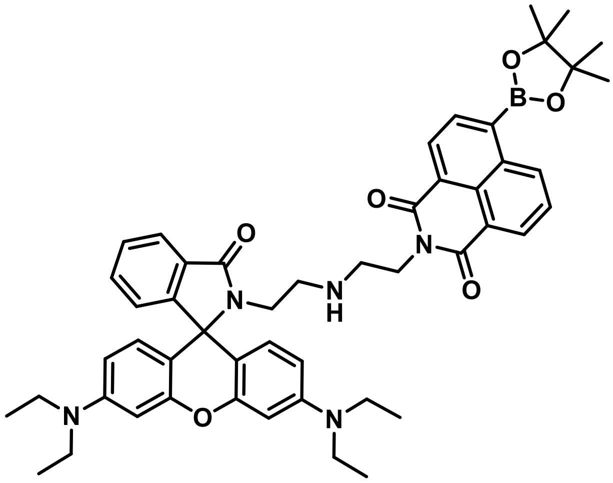

ATP-LW

ATP-LW was synthesized using revised reported literature procedures.[22] ^{1}H NMR (500 MHz, $CDCl_3$) δ_H 9.08 (d, *J* = 8.5 Hz, 1H, Ar*H*), 8.52 (dd, *J* = 7.3, 1.2 Hz,1H, Ar*H*), 8.49 (d, *J* = 7.2 Hz, 1H, Ar*H*), 8.24 (d, *J* = 7.3 Hz, 1H, Ar*H*), 7.73 (dd, *J* = 8.5, 7.3 Hz, 1H, Ar*H*), 7.70-7.61 (m, 1H, Ar*H*), 7.42-7.35 (m, 2H, Ar*H*), 7.04 (d, *J* = 7.6 Hz, 1H, Ar*H*), 6.43 (d, *J* = 8.9 Hz, 2H, Ar*H*), 6.35 (d, *J* = 2.6 Hz, 2H, Ar*H*), 6.26 (dd, *J* = 8.9, 2.6 Hz, 2H, Ar*H*), 4.22 (bs, 2H, $[C(O)]_2NCH_2CH_2$), 3.38-3.23 (m, 10H, 4 x CH_3CH_2 + $C(O)NCH_2$), 2.85 (bs, 2H, $NHCH_2CH_2$), 2.49 (bs, 2H, $NHCH_2CH_2$), 1.45 (s, 12H, 4 x $OCCH_3$), 1.134 (t, 12H *J* = 7.0 Hz, 4 x NCH_2CH_3). ^{13}C NMR (126 MHz, $CDCl_3$) δ_C 168.7, 164.6, 164.6, 153.8, 153.4, 148.9, 135.8, 135.4, 135.0, 132.5, 131.0, 129.9, 128.8, 128.1, 128.0, 127.1, 124.9, 123.9, 122.9, 122.8, 108.3, 97.9, 84.7, 65.2, 47.7, 47.1, 44.5, 39.8, 38.4, 25.1, 12.7. HRMS (ES^+): calc. for $C_{50}H_{56}BN_5O_6$ $[M+H]^+$ m/z 834.4405, found m/z 834.4407.

4. Generation of various ROS/RNS

ROO•

ROO• was generated from 2,2'-azobis(2-amidinopropane) dihydrochloride (AAPH). AAPH was added into deionized water, and then stirred at 37 °C for 30 min to give 0.1 M AAPH.

$O_2^{\bullet -}$

Superoxide was generated from KO_2. KO_2 and 18-crown-6 ether (2.5 eq) was dissolved in DMSO to afford a 0.25 M solution.

•OH

Hydroxyl radical was generated by the Fenton reaction. To prepare an •OH solution, hydrogen peroxide (H_2O_2, 10 eq) was added to $Fe(ClO_4)_2$ in deionized water.

$^1O_2$

$^1O_2$ was generated by reacting H_2O_2 (1 mM) with NaClO (1 mM). The solution of H_2O_2 was added in one portion to the aqueous solution of NaClO and stirred for 2 minutes. The resulting solution was used immediately.

$ONOO^-$

Simultaneously, 0.6 M $NaNO_2$, 0.6 M in HCl, 0.7 M in H_2O_2 was added at to a 3 M NaOH solution at 0 ºC. The concentration of peroxynitrite was estimated using an extinction co-efficient of 1670 $M^{-1}cm^{-1}$ at 302 nm in 0.5 M sodium hydroxide aqueous solution.

ClO^-

The concentration of ClO^- was determined from the absorption at 292 nm (ε = 350 M^{-1} cm^{-1}).

H_2O_2

The concentration of H_2O_2 was determined from the absorption at 240 nm (ε = 43.6 M^{-1} cm^{-1}).

5. UV-Vis and fluorescence analyses

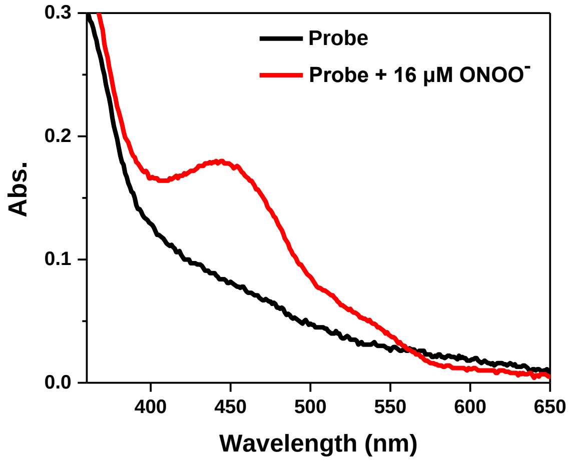

Figure S1. Absorption spectra of probe **ATP-LW** (15 μM) with and without $ONOO^-$ (16 μM) in modified PBS buffer solution (10 mM, V/V, EtOH/H_2O = 1/99, pH = 7.40) after 1 min.

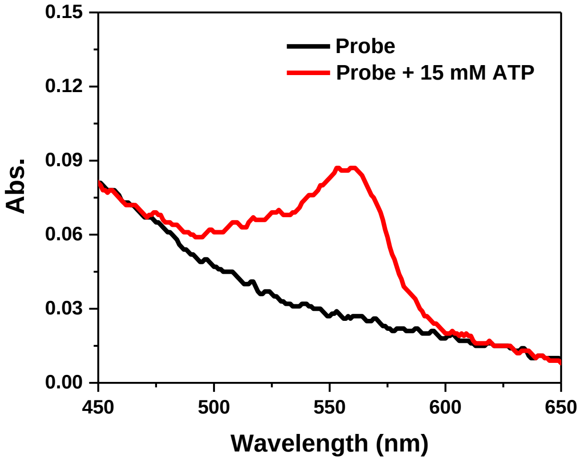

Figure S2. Absorption spectra of probe **ATP-LW** (15 μM) with and without ATP (15 mM) in modified PBS buffer solution (10 mM, V/V, EtOH/H_2O = 1/99, pH = 7.40) after 100 min.

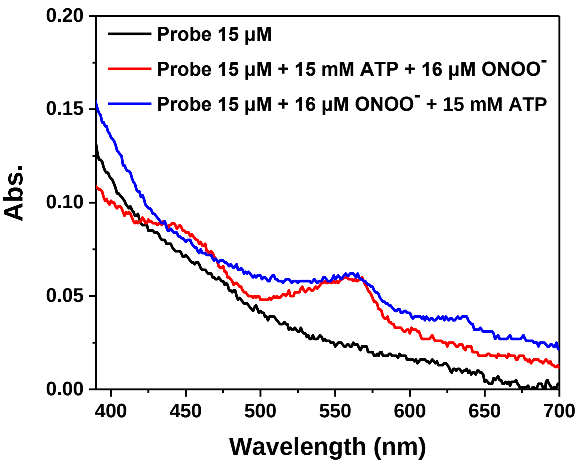

Figure S3. Absorption spectra of probe **ATP-LW** (15 μM), **ATP-LW** (15 μM) with addition of ATP (15 mM) wait 100 min prior to the addition of $ONOO^-$ (16 μM) wait 1 min, **ATP-LW** (15 μM) with addition of $ONOO^-$ (16 μM) wait 1 min then add ATP (15 mM) and wait 100 min in modified PBS buffer solution (10 mM, V/V, EtOH/H_2O = 1/99, pH = 7.40).

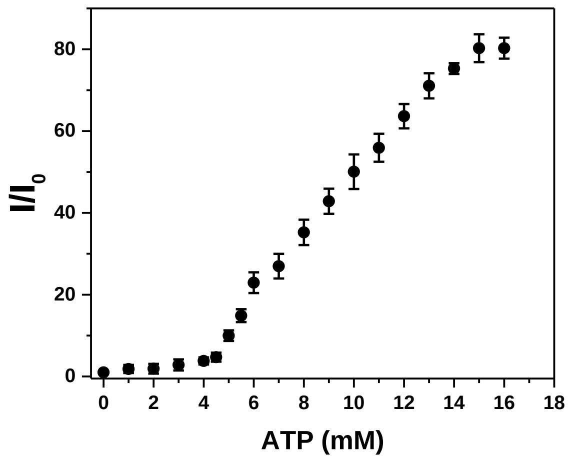

Figure S4. Emission at 587 nm of the probe **ATP-LW** (15 μM) as a function of increasing ATP concentration (from 0 to 16 mM) in modified PBS buffer solution (10 mM, V/V, EtOH/H_2O = 1/99, pH = 7.40) after 100 min. Excited at 520 (bandwidth 8) nm.

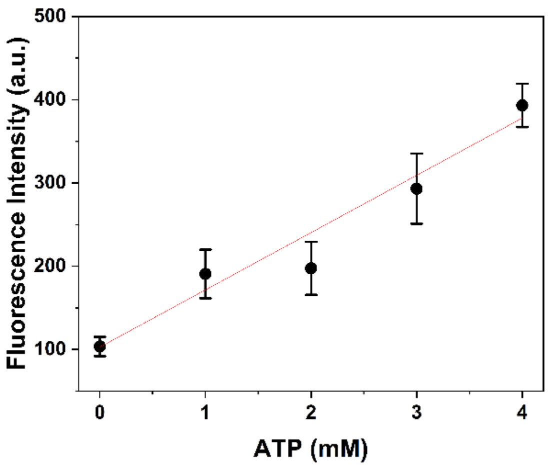

Figure S5. Relationship between fluorescence intensity at at 587 nm of **ATP-LW** (15 μM) and concentration of ATP (0 – 4 mM). $\lambda_{ex/em}$ = 520/587 nm. Linear equation: $y_1 = 102.747 + 68.812\times$ [ATP] (mM), $R^2 = 0.976$. On this basis, the limit of detection is 63 μM.

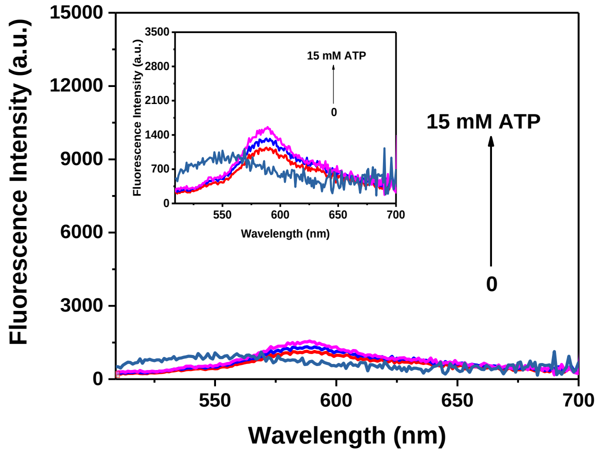

Figure S6. Changes in the fluorescence emission intensity of probe **ATP-LW** (15 μM) seen as a function of increasing ATP concentration (from 0 to 15 mM) in modified PBS buffer solution (10 mM, V/V, EtOH/H_2O = 1/99, pH = 7.40) after 100 min. Inset: enlarged spectra. Excited at 488 (bandwidth 8) nm.

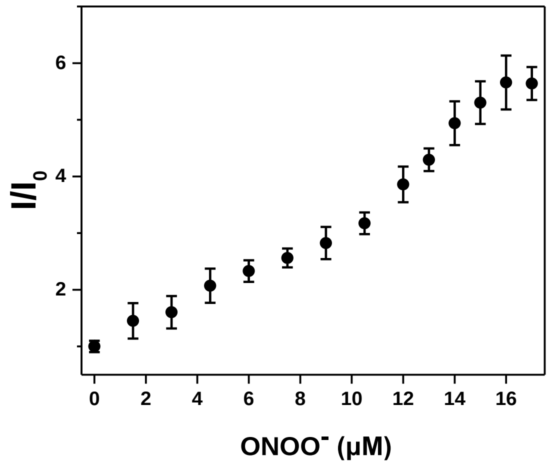

Figure S7. Emission at 562 nm of the probe **ATP-LW** (15 μM) as a function of increasing ONOO$^-$ concentration (from 0 to 17 μM) in modified PBS buffer solution (10 mM, V/V, EtOH/H_2O = 1/99, pH = 7.40) after 1 min. Excited at 450 (bandwidth 8) nm.

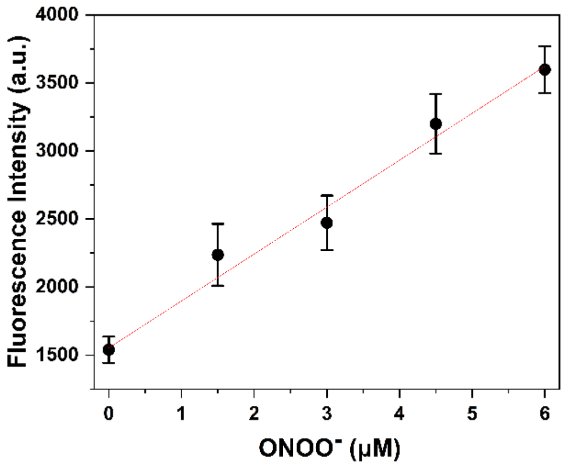

Figure S8. Relationship between fluorescence intensity at 562 nm of **ATP-LW** (15 μM) and concentration of ONOO$^-$ (0 − 6 μM). $\lambda_{ex/em}$ = 450/562 nm. Linear equation: y_2 = 1553.170 + 344.790 × [ONOO$^-$] (μM), R^2 = 0.992. On this basis, the limit of detection is 26 nM.

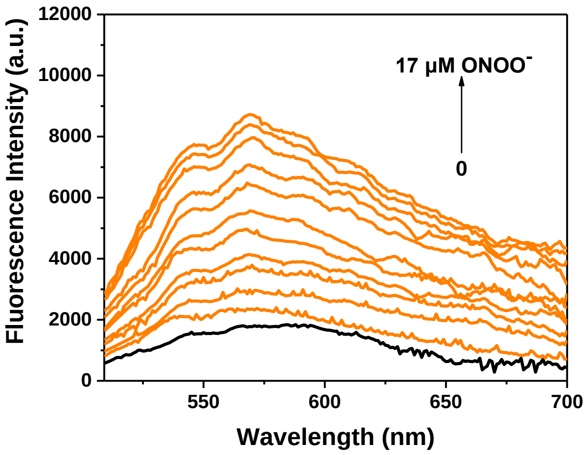

Figure S9. Changes in fluorescence emission intensity of the probe **ATP-LW** (15 μM) as a function of increasing ONOO⁻ concentration (from 0 to 17 μM) in modified PBS buffer solution (10 mM, V/V, EtOH/H_2O = 1/99, pH = 7.40) after 1 min. Excited at 488 (bandwidth 8) nm.

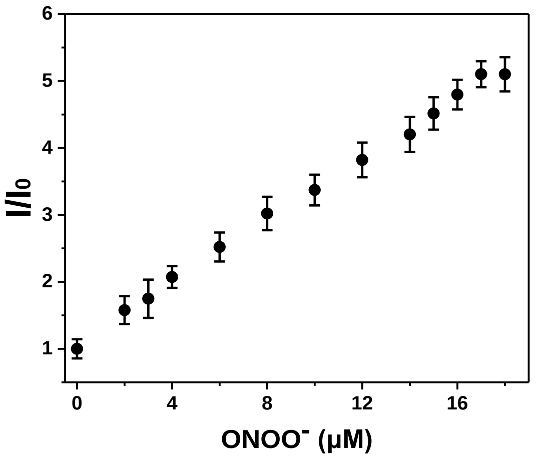

Figure S10. Emission at 568 nm of the probe **ATP-LW** (15 μM) as a function of increasing ONOO⁻ concentration (from 0 to 18 μM) in modified PBS buffer solution (10 mM, V/V, EtOH/H_2O = 1/99, pH = 7.40) after 1 min. Excited at 488 (bandwidth 8) nm.

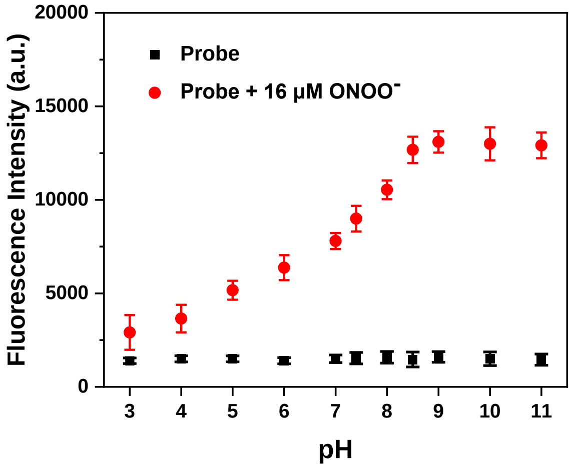

Figure S11. Effects of pH on the fluorescence of the probe **ATP-LW** (15 μM) in the absence and presence of $ONOO^-$ (16 μM). $\lambda_{ex/em}$ = 450 (bandwidth 8) nm /562 nm.

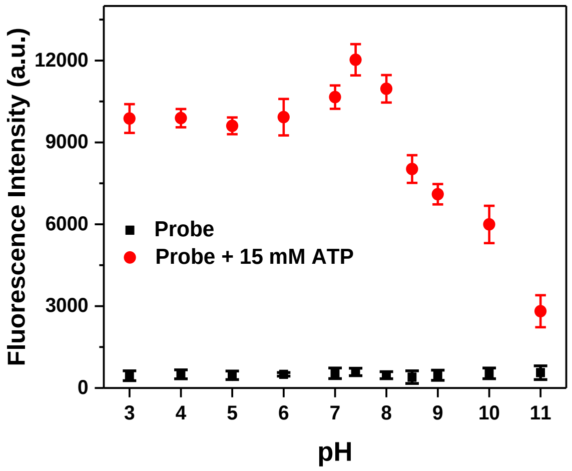

Figure S12. Effects of pH on the fluorescence of the probe **ATP-LW** (15 μM) in the absence and presence of ATP (15 mM). $\lambda_{ex/em}$ = 520 (bandwidth 8) nm /587 nm.

Experimental procedure for Figure S13: A solution of $ONOO^-$ (16 μM) was added into **ATP-LW** (15 μM), and the fluorescence spectrum (curve 2, in orange) was recorded after 1 min. Then various concentrations of ATP (1 – 15 mM) were added to the solution, and each line of the fluorescence spectra (curves 3-13, in pink) was recorded after 100 min. λ_{ex} = 520 (bandwidth 8) nm.

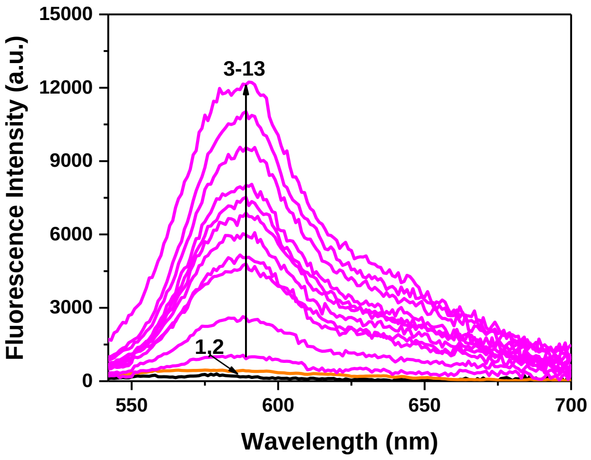

Figure S13. Fluorescence spectra of **ATP-LW** (15 μM, curve 1, black) as a function of increasing $ONOO^-$ concentration (16 μM, curve 2, orange) after 1 min followed by the addition of ATP (1 – 15 mM) (curves 3-13) in modified PBS buffer solution (10 mM, V/V, EtOH/H_2O = 1/99, pH = 7.40) after 100 min. λ_{ex} = 520 (bandwidth 8) nm.

Experimental procedure for Figure S14: A solution of ATP (15 mM) was added into **ATP-LW** (15 μM), and the fluorescence spectrum (curve 2, in pink) was recorded after 100 min. Then various concentrations of $ONOO^-$ (1 – 15 μM) were added to the solution, and each line of the fluorescence spectra (curves 3 – 15, in orange) was recorded after 1 min. λ_{ex} = 450 (bandwidth 8) nm.

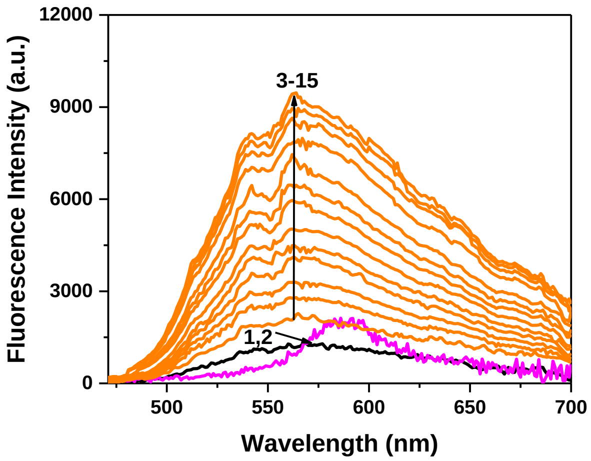

Figure S14. Fluorescence spectra of **ATP-LW** (15 μM, curve 1, black) as a function of increasing ATP concentration (15 mM, curve 2, pink) after 100 min followed by the addition of $ONOO^-$ (1 – 15 μM) (curves 3 – 15) in modified PBS buffer solution (10 mM, V/V, EtOH/H_2O = 1/99, pH = 7.40) after 1 min. λ_{ex} = 450 (bandwidth 8) nm.

Experimental procedure for Figure S15: A solution of ATP (15 mM) was added into **ATP-LW** (15 μM), and the fluorescence spectrum (curve 2, in pink) was recorded after 100 min. Then various concentrations of $ONOO^-$ (1 – 17 μM) were added to the solution, each line of the fluorescence spectra (curves 3 – 12, in orange) was recorded after 1 min. λ_{ex} = 488 (bandwidth 8) nm.

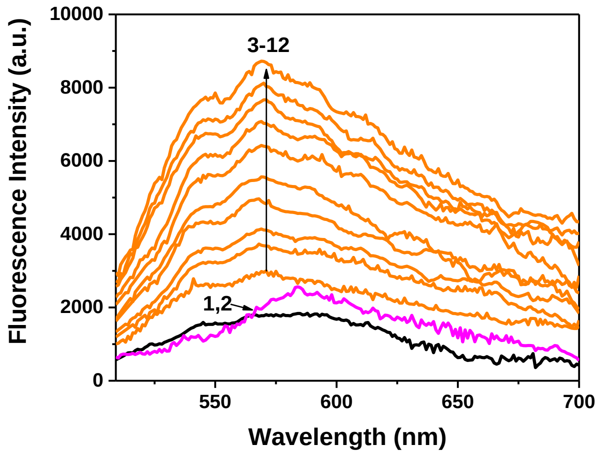

Figure S15. Fluorescence spectra of **ATP-LW** (15 μM, curve 1, black) as a function of increasing ATP concentration (15 mM, curve 2, pink) after 100 min followed by the addition of $ONOO^-$ (1 – 17 μM) (curves 3 – 12) in modified PBS buffer solution (10 mM, V/V, EtOH/H_2O = 1/99, pH = 7.40) after 1 min. λ_{ex} = 488 (bandwidth 8) nm.

Experimental procedure for Figure S16: A solution of $ONOO^-$ (16 μM) in modified PBS buffer solution (10 mM, V/V, EtOH/H_2O = 1/99, pH = 7.40) was placed in a Greiner Bio-One microplate (96-well, PS, f-bottom (chimney well), black-walled), and the intensity was measured every 0.21 s for 2.95 s. A solution of **ATP-LW** (15 μM) was then pumped into this solution, and the fluorescence spectrum was then recorded every 0.21 s from 4.15 s to 29.22 s.

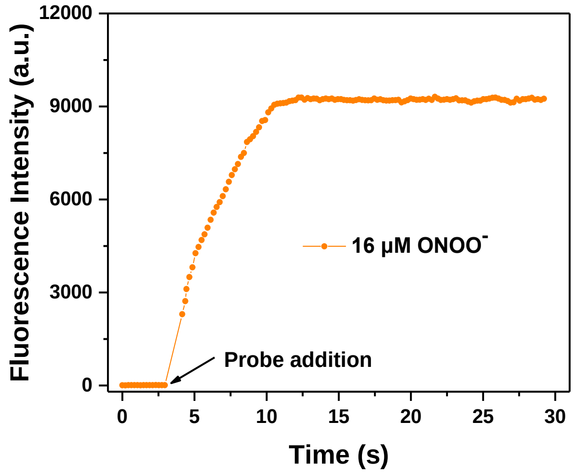

Figure S16. Plots of fluorescence intensity *vs.* the reaction time of the probe **ATP-LW** (15 μM) with 16 μM $ONOO^-$ ($\lambda_{ex/em}$ = 450 (bandwidth 8) nm /562 nm) in modified PBS buffer solution (10 mM, V/V, EtOH/H_2O = 1/99, pH = 7.40).

Experimental procedure for Figure S17: A solution of ATP (15 mM) in modified PBS buffer solution (10 mM, V/V, EtOH/H_2O = 1/99, pH = 7.40) was placed in a Greiner Bio-One microplate (96-well, PS, f-bottom (chimney well), black-walled), and the intensity was measured every 5 min for 15 min. A solution of **ATP-LW** (15 μM) was then pumped into this solution, and the fluorescence spectrum recorded every 5 min from 20 min to 165 min.

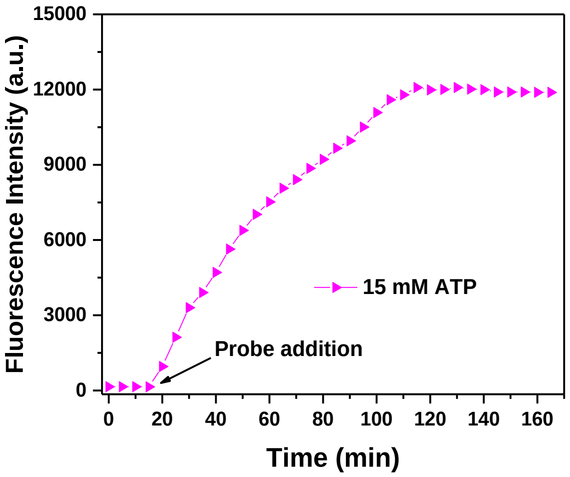

Figure S17. Plots of fluorescence intensity *vs.* the reaction time of the probe **ATP-LW** (15 μM) with 15 mM ATP disodium salt ($\lambda_{ex/em}$ = 520 (bandwidth 8) nm /587 nm) in modified PBS buffer solution (10 mM, V/V, EtOH/H_2O = 1/99, pH = 7.40).

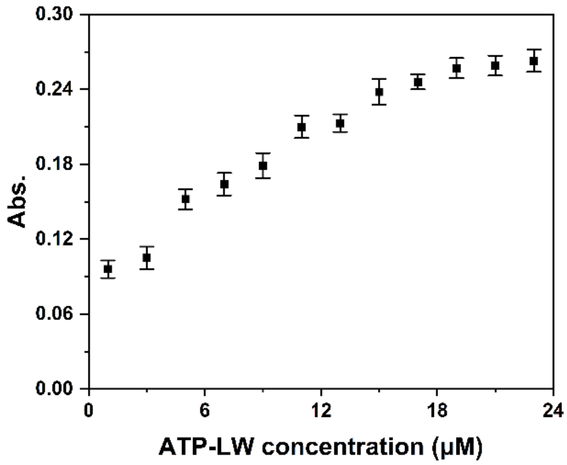

Figure S18. Absorption intensity of various concentrations of **ATP-LW** (1-23 μM) with $ONOO^-$ (16 μM) in PBS buffer solution (10 mM, V/V, EtOH/H_2O = 1/99, pH = 7.40) recorded after 1 min, $\lambda_{abs.}$ = 445 nm.

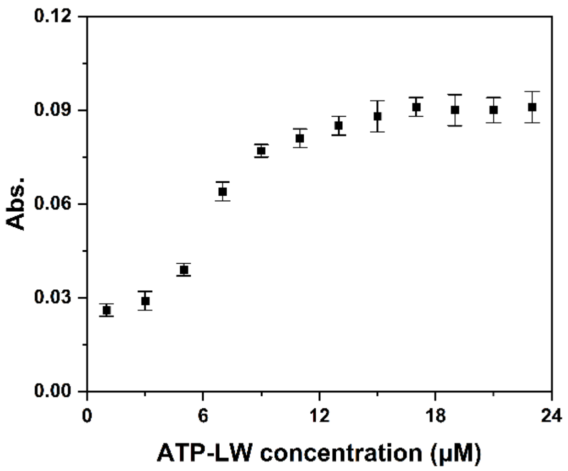

Figure S19. Absorption intensities of various concentrations of **ATP-LW** (1-23 μM) with ATP (15 mM) in PBS buffer solution (10 mM, V/V, EtOH/H_2O = 1/99, pH = 7.40) recorded after 100 min, $\lambda_{abs.}$ = 553 nm.

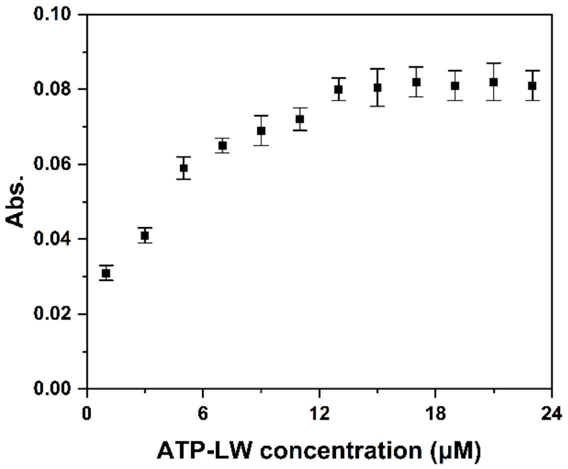

Figure S20. Absorption intensities of various concentrations of **ATP-LW** (1-23 μM) pre-incubated with $ONOO^-$ (16 μM) for 1 min, followed by addition of ATP (15 mM) and waiting 100 min before determining the spectral intensity in PBS buffer solution (10 mM, V/V, EtOH/H_2O = 1/99, pH = 7.40); $\lambda_{abs.}$ = 553 nm.

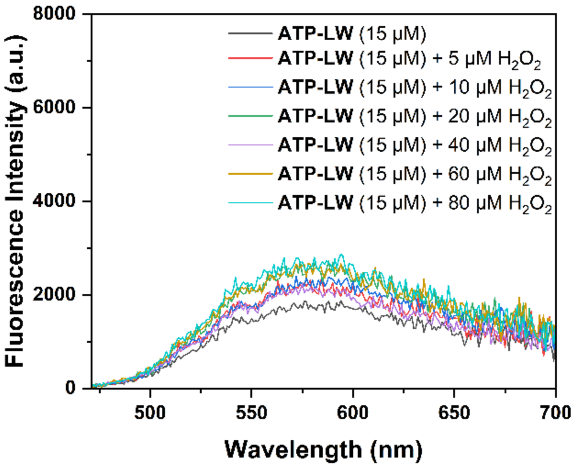

Figure S21. Changes in the fluorescence emission intensity of the probe **ATP-LW** (15 μM) as a function of increasing H_2O_2 (from 0 to 80 μM) in PBS buffer solution (10 mM, V/V, EtOH/H_2O = 1/99, pH = 7.40) as recorded after 15 min. Excited at 450 (bandwidth 8) nm.

6. Theoretical calculations to confirm absence of FRET

Quantum Calculations Method

All calculations of geometric structure and electronic spectra were performed using the Gaussian 16 program.[23] The functional ωB97X-D and the TZVP basis set[24-26] were used in these density functional theoretical (DFT) calculations.[27] The electronic transition energies and corresponding oscillator strengths were calculated by time-dependent density functional theory (TD-DFT) at the ωB97X-D/TZVP level. Water was used as solvent in the CPCM calculations to account for solvent effects.[28]

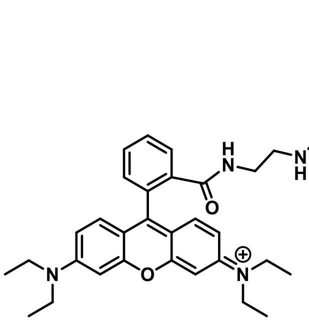

XT-LW

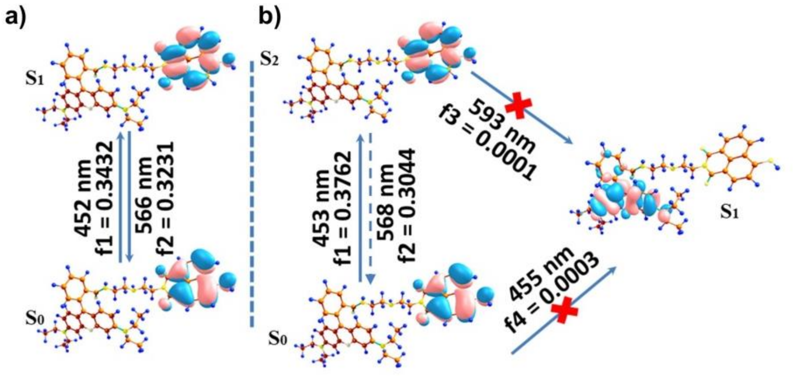

Figure S22. Density functional theoretical calculations of the product **XT-LW**. (a) Molecular optimal configuration of **XT-LW** at ground state (S_0) and the first excited singlet state (S_1), where S_0 and S_1 are defined by using the 4-hydroxy-1,8-naphthimide fragment as the region of interest (ROI) according to the experimental spectral results. The theoretical absorption and emission wavelengths of **XT-LW** are 452 nm and 566 nm, respectively, which is in agreement with the experimental spectral results (**Figure S1** and **S2**). (b) Optimized configuration of **XT-LW** to confirm no FRET between the two fluorophores. Transitions from the S_2 to the S_1 and the S_0 to the S_1 are forbidden (oscillator strength $f < 0.001$). In this case, the rhodamine fragment is the ROI to define S_1, and the 4-hydroxy-1,8-naphthimide fragment is the ROI to define S_0 and the second excited singlet state (S_2).

Molecular internal coordinates

C	9.115606000000	2.218460000000	-1.125342000000
C	10.043258000000	1.227224000000	-0.891621000000
C	9.699329000000	0.070662000000	-0.135596000000
C	8.382515000000	-0.030106000000	0.366387000000
C	7.444786000000	0.993936000000	0.116398000000
C	7.819085000000	2.095640000000	-0.620660000000

C	8.019196000000	-1.170344000000	1.112609000000
C	8.933314000000	-2.169758000000	1.349463000000
C	10.241829000000	-2.065793000000	0.852705000000
C	10.619656000000	-0.967088000000	0.122774000000
C	6.080042000000	0.883860000000	0.635557000000
N	5.777985000000	-0.234674000000	1.410433000000
C	6.647377000000	-1.291867000000	1.639479000000
O	6.270349000000	-2.272319000000	2.261057000000
O	11.306066000000	1.290114000000	-1.357131000000
O	5.218782000000	1.723558000000	0.421162000000
H	9.401029000000	3.088762000000	-1.702851000000
H	7.097206000000	2.879238000000	-0.810086000000
H	8.636071000000	-3.037372000000	1.923614000000
H	10.951240000000	-2.858266000000	1.047687000000
H	11.627602000000	-0.887388000000	-0.261434000000
H	11.436606000000	2.105544000000	-1.862123000000
C	-6.605435000000	-1.271803000000	-0.102566000000
C	-5.483284000000	-0.435293000000	-0.348213000000
C	-5.605378000000	0.908602000000	0.079176000000
C	-6.738973000000	1.411552000000	0.668389000000
C	-7.849372000000	0.569603000000	0.895874000000
C	-7.736946000000	-0.801152000000	0.479321000000
O	-4.572041000000	1.771329000000	-0.065325000000
C	-3.407366000000	1.387156000000	-0.638834000000
C	-3.249845000000	0.067039000000	-1.122623000000
C	-4.288706000000	-0.857077000000	-0.942944000000
C	-2.007175000000	-0.221677000000	-1.749118000000
C	-1.015319000000	0.700619000000	-1.836890000000
C	-1.180192000000	2.026829000000	-1.308854000000
C	-2.418698000000	2.337848000000	-0.706372000000
C	-4.112437000000	-2.267432000000	-1.370752000000
C	-3.142734000000	-3.082351000000	-0.773963000000
C	-2.957937000000	-4.383845000000	-1.222585000000
C	-3.738311000000	-4.883958000000	-2.256417000000
C	-4.699117000000	-4.079123000000	-2.850317000000
C	-4.885370000000	-2.775987000000	-2.409529000000
N	-8.963961000000	1.031806000000	1.493369000000
C	-10.178718000000	0.214797000000	1.537022000000
C	-10.926534000000	0.222047000000	0.213850000000
C	-9.074962000000	2.482092000000	1.729361000000
C	-10.338655000000	2.922277000000	2.438447000000
N	-0.198159000000	2.942795000000	-1.398214000000
C	1.132242000000	2.558727000000	-1.873724000000
C	1.947243000000	1.831804000000	-0.815789000000

C	-0.365294000000	4.215237000000	-0.674473000000
C	0.775259000000	5.198507000000	-0.833740000000
C	-2.334707000000	-2.537078000000	0.364562000000
O	-2.879124000000	-1.992574000000	1.328323000000
N	-1.008982000000	-2.652686000000	0.258241000000
H	-6.555176000000	-2.313040000000	-0.390920000000
H	-6.735413000000	2.451967000000	0.950853000000
H	-8.559490000000	-1.482599000000	0.624329000000
H	-1.850131000000	-1.202073000000	-2.179605000000
H	-0.093563000000	0.418171000000	-2.319935000000
H	-2.629612000000	3.310097000000	-0.290937000000
H	-2.212974000000	-5.011737000000	-0.750350000000
H	-3.595576000000	-5.901964000000	-2.593985000000
H	-5.306204000000	-4.462321000000	-3.659921000000
H	-5.625841000000	-2.140419000000	-2.879198000000
H	-9.916245000000	-0.797110000000	1.835649000000
H	-10.811013000000	0.600230000000	2.330284000000
H	-11.830606000000	-0.382768000000	0.291455000000
H	-10.306867000000	-0.184030000000	-0.587425000000
H	-11.213972000000	1.239673000000	-0.056259000000
H	-8.219414000000	2.787726000000	2.332329000000
H	-8.992896000000	2.999758000000	0.768308000000
H	-10.276548000000	4.001108000000	2.581098000000
H	-10.434063000000	2.458630000000	3.420384000000
H	-11.237298000000	2.712407000000	1.858788000000
H	1.023608000000	1.949409000000	-2.767972000000
H	1.646471000000	3.459519000000	-2.192602000000
H	2.915748000000	1.546212000000	-1.227774000000
H	1.433000000000	0.928377000000	-0.481472000000
H	2.115285000000	2.472057000000	0.052280000000
H	-1.280576000000	4.687547000000	-1.033815000000
H	-0.513458000000	3.994209000000	0.387178000000
H	0.517870000000	6.094526000000	-0.268931000000
H	0.918840000000	5.487989000000	-1.874739000000
H	1.715465000000	4.812957000000	-0.439909000000
C	-0.119399000000	-1.954337000000	1.172114000000
H	-0.627097000000	-3.020909000000	-0.603047000000
C	1.275450000000	-1.888541000000	0.586224000000
H	-0.505172000000	-0.944410000000	1.331230000000
H	-0.102514000000	-2.460826000000	2.140338000000
N	2.167454000000	-1.189161000000	1.500177000000
H	1.628493000000	-2.903551000000	0.356401000000
H	1.243070000000	-1.336582000000	-0.357507000000
C	3.508321000000	-1.070619000000	0.944243000000

H	2.228420000000	-1.733384000000	2.357547000000
C	4.413258000000	-0.353028000000	1.930150000000
H	3.944720000000	-2.047529000000	0.695963000000
H	3.450862000000	-0.493031000000	0.018756000000
H	4.043380000000	0.648973000000	2.127066000000
H	4.462710000000	-0.898756000000	2.869442000000

7. Transient absorption studies confirming the absence of electron transfer

NA-OH: **ATP-LW** (0.6 mmol/L) + $ONOO^-$ (60 μmol/L, 1 min)

Rh-Bpin: **ATP-LW** (0.6 mmol/L) + ATP (7 mmol/L, 100 min)

XT-LW: **ATP-LW** (0.6 mmol/L) + ATP (7 mmol/L, 100 min) + $ONOO^-$ (60 μmol/L, 1 min)

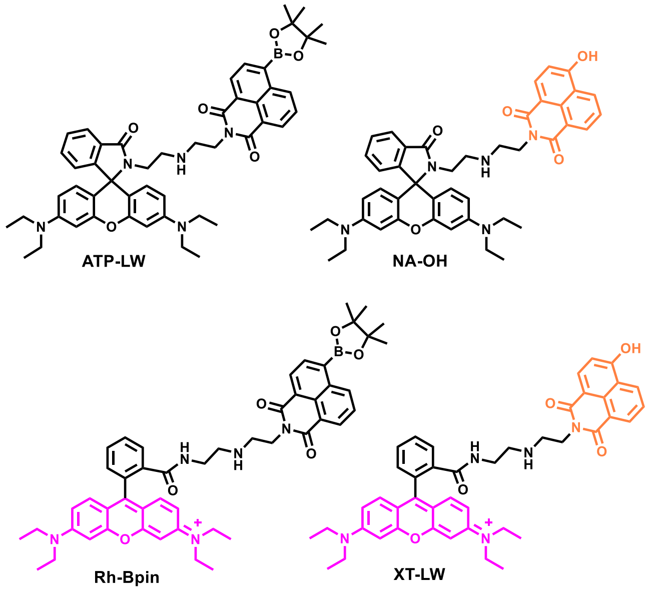

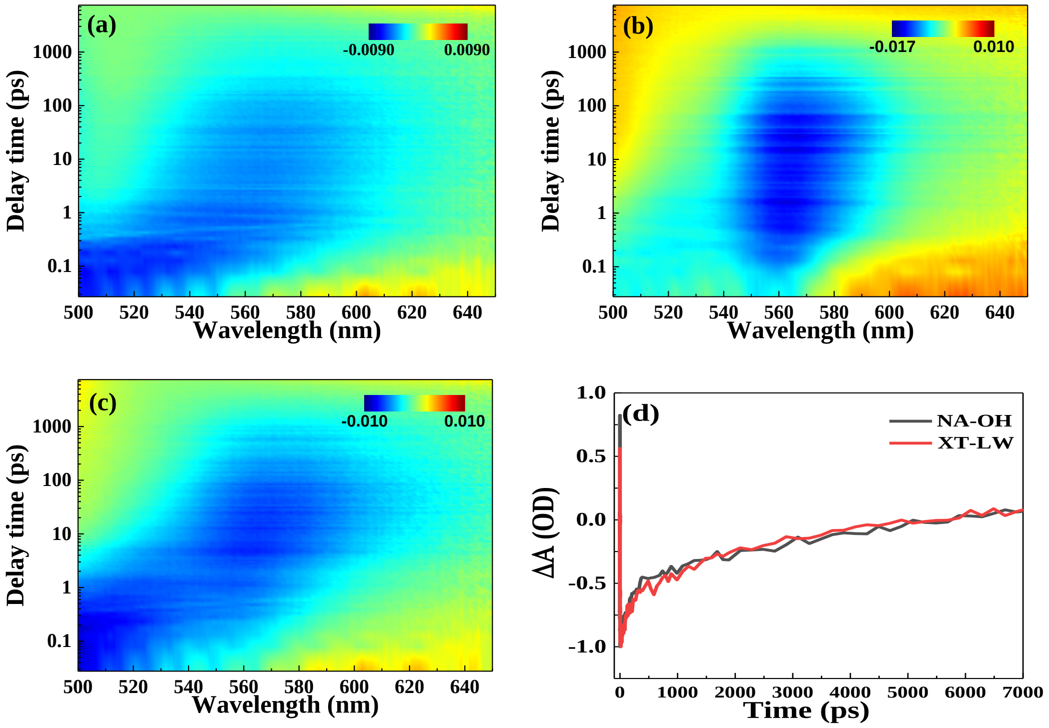

Figure S23. Time- and wavelength-dependent transient absorbance (TA) color maps of **ATP-LW** (600 μM) recorded 1 min after the addition of $ONOO^-$ (60 μM) (a), **ATP-LW** (600 μM) after adding ATP (7 mM) and waiting 100 min (b), **ATP-LW** (600 μM) after adding ATP (7 mM) and waiting 100 min prior to the addition of $ONOO^-$ (60 μM) and waiting 1 min (c). Studies were carried out in PBS/acetonitrile (v:v = 1:1, PBS 10 mM, pH = 7.40); solutions pumped at 400 nm. (d) TA attenuation curves corresponding to panels a and c at 572 nm.

As can be seen from Figure S23 (a-c), similar fluorescence signatures are seen for **NA-OH** (**ATP-LW** (600 μM) determined 1 min after the addition of $ONOO^-$ (60 μM) wait 1 min) and for **XT-LW** (**ATP-LW** (600 μM) after the addition of ATP (7 mM), waiting 100 min before adding $ONOO^-$ (60 μM), and waiting 1 min to carry out the measurement. These signatures differ from those of **Rh-Bpin** (**ATP-LW** (600 μM) seen upon the addition of ATP (7 mM) and waiting 100 min to make the measurement. In addition, the dynamic curves (Fig. S23d) show that the signal attenuation curve of **NA-OH** at 572 nm is consistent with that of **XT-LW.** These results lead us to infer that there is no electron transfer from the naphthalimide subunit to rhodamine in **XT-LW**.

8. Mass spectrometric analyses

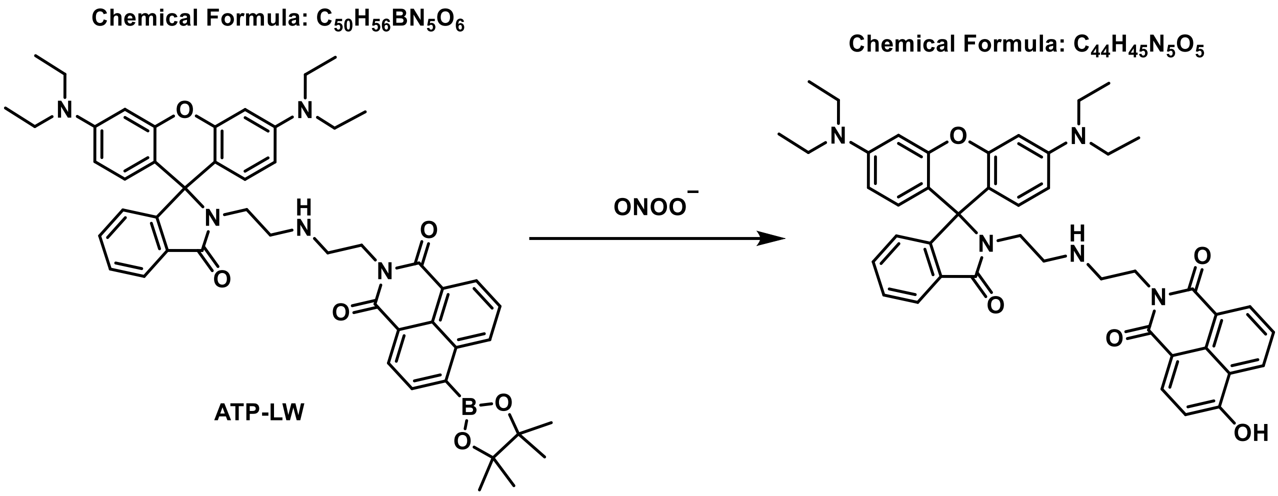

Scheme S3. Proposed reaction between probe **ATP-LW** and $ONOO^-$.

Probe **ATP-LW** (5 mM, in DMSO) was diluted to 1 μM in water (Figure S24), then $ONOO^-$ (2 μM) was added (Figure S25).

Walkup MS Report

**Data File**	probe_Pos_5mins_MS_16182.d	**Sample Name**	probe
**Sample Type**	Sample	**Position**	P1-A6
**Instrument Name**	6545 QTof	**User Name**	Xue Tian
**Acq Method**	Pos_5mins_MS.m	**Acquired Time**	17/02/2021 17:26:58
**IRM Calibration Status**	Success	**DA Method**	Pos_5mins_MS.m
**Comment**			

**Sample Group**		**Info.**	
**Walkup Sample Description**		**Walkup Method**	Pos_5Mins_C18
**Formula**	C50H56BN5O6	**Walkup Method Description**	Positive mode ionization using C18 column chromatography
**Stream Name**	LC 1	**Acquisition SW Version**	6200 series TOF/6500 series Q-TOF B.09.00 (B9044.0)

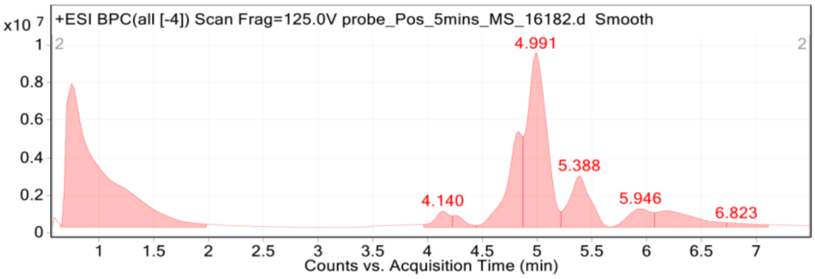

Figure 1: Base peak chromatogram

User Chromatogram Peak List

RT (min)	Area	Area %	Area Sum (%)	Base Peak (m/z)	Width (min)
0.75	172405396	100.00	40.54	102.1310	0.296
4.14	8164761	4.74	1.92	185.1194	0.136
4.26	4836815	2.81	1.14	185.1193	0.126
4.83	49970070	28.98	11.75	752.3743	0.134
4.99	112419298	65.21	26.43	834.4552	0.194
5.39	35990489	20.88	8.46	443.3425	0.180
5.95	14730643	8.54	3.46	125.9897	0.228
6.19	22688600	13.16	5.33	141.9625	0.353
6.82	4112857	2.39	0.97	922.0228	0.254

Compound Table

Compound Label	RT (min)	Observed mass (m/z)	Neutral observed mass (Da)	Theoretical mass (Da)	Mass error (ppm)	Isotope match score (%)
Cpd 1: C50 H56 B N5 O6	4.98	834.4407	832.4358	832.4360	-0.21	99.34

Mass errors of between -5.00 and 5.00 ppm with isotope match scores above 60% are considered confirmation of molecular formulae

Walkup MS Report

Compound specific information

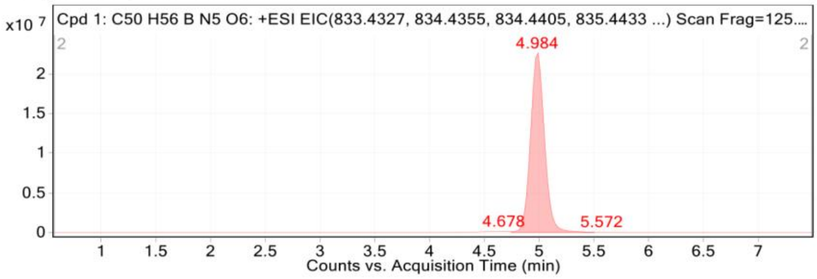

Figure: Extracted ion chromatogram (EIC) of compound.

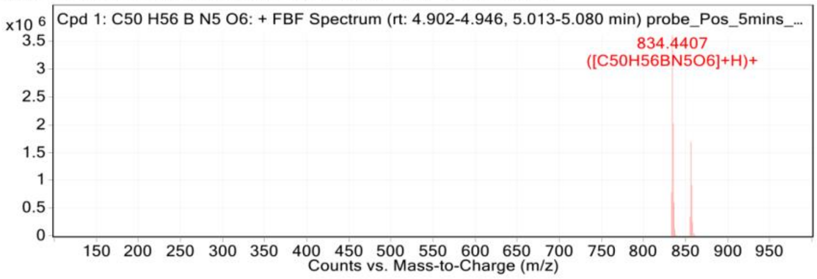

Figure: Full range view of Compound spectra and potential adducts.

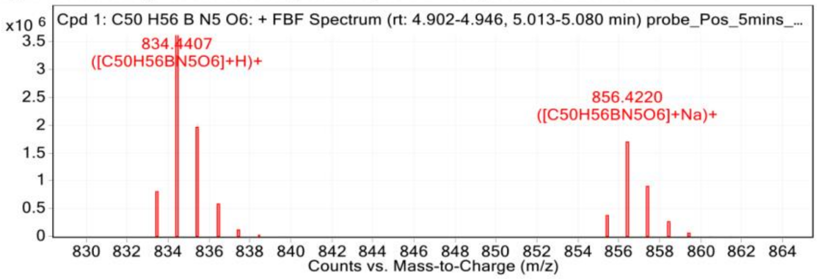

Figure: Zoomed Compound spectra view
(red boxes indicating expected theoretical isotope spacing and abundance)

Compound isotope peak List

m/z	z	Abund	Formula	Ion
833.4427	1	781395.0	C50H56BN5O6	(M+H)+
834.4407	1	3620850.0	C50H56BN5O6	(M+H)+
835.4430	1	2019427.8	C50H56BN5O6	(M+H)+
836.4457	1	597028.3	C50H56BN5O6	(M+H)+
837.4485	1	113882.2	C50H56BN5O6	(M+H)+
855.4240	1	344530.7	C50H56BN5O6	(M+Na)+
856.4220	1	1689579.3	C50H56BN5O6	(M+Na)+
857.4248	1	915320.8	C50H56BN5O6	(M+Na)+
858.4279	1	248062.0	C50H56BN5O6	(M+Na)+
859.4297	1	52547.4	C50H56BN5O6	(M+Na)+

--- End Of Report ---

Figure S24. LC-MS of **ATP-LW**.

Walkup MS Report

**Data File**	probe+ onoo_Pos_5mins_MS_16163.d	**Sample Name**	probe+ onoo
**Sample Type**	Sample	**Position**	P1-A8
**Instrument Name**	6545 QTof	**User Name**	Xue Tian
**Acq Method**	Pos_5mins_MS.m	**Acquired Time**	17/02/2021 13:48:15
**IRM Calibration Status**	Success	**DA Method**	Pos_5mins_MS.m
**Comment**			

**Sample Group**		**Info.**	
**Walkup Sample Description**		**Walkup Method**	Pos_5Mins_C18
**Formula**	C44H45N5O5	**Walkup Method Description**	Positive mode ionization using C18 column chromatography
**Stream Name**	LC 1	**Acquisition SW Version**	6200 series TOF/6500 series Q-TOF B.09.00 (B9044.0)

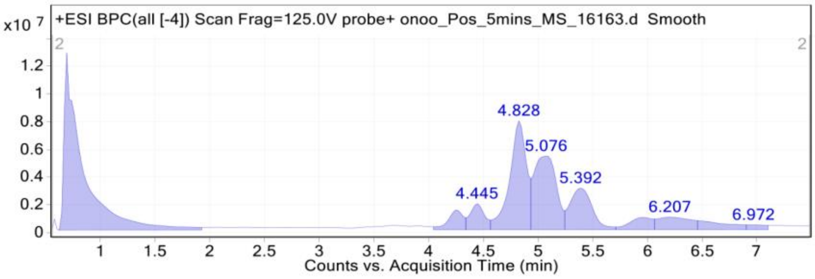

Figure 1: Base peak chromatogram

User Chromatogram Peak List

RT (min)	Area	Area %	Area Sum (%)	Base Peak (m/z)	Width (min)
0.70	150450217	100.00	34.79	101.0065	0.150
4.26	13784563	9.16	3.19	185.1189	0.145
4.45	17231152	11.45	3.98	442.2563	0.140
4.83	83984528	55.82	19.42	724.3612	0.158
5.08	75543142	50.21	17.47	952.5616	0.226
5.39	42642889	28.34	9.86	443.3420	0.219
5.96	13102935	8.71	3.03	125.9896	0.225
6.21	19124430	12.71	4.42	141.9624	0.312
6.57	12575443	8.36	2.91	922.0219	0.300
6.97	4062947	2.70	0.94	922.0218	0.165

Compound Table

Compound Label	RT (min)	Observed mass (m/z)	Neutral observed mass (Da)	Theoretical mass (Da)	Mass error (ppm)	Isotope match score (%)
Cpd 1: C44 H45 N5 O5	4.83	724.3490	723.3419	723.3421	-0.22	99.42

Mass errors of between -5.00 and 5.00 ppm with isotope match scores above 60% are considered confirmation of molecular formulae

Walkup MS Report

Compound specific information

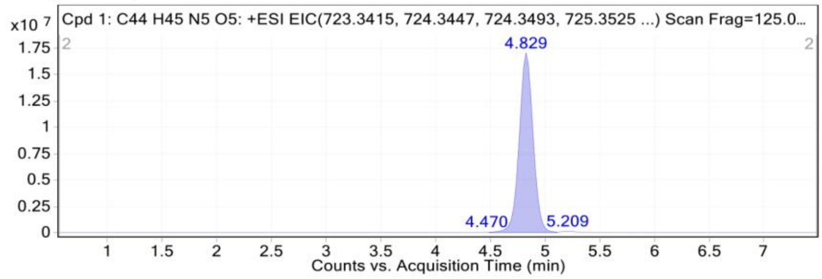

Figure: Extracted ion chromatogram (EIC) of compound.

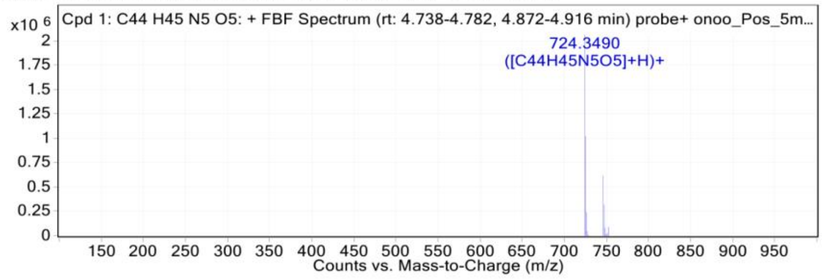

Figure: Full range view of Compound spectra and potential adducts.

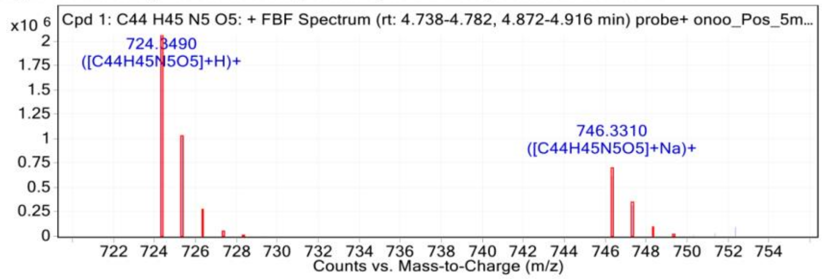

Figure: Zoomed Compound spectra view
(red boxes indicating expected theoretical isotope spacing and abundance)

Compound isotope peak List

m/z	z	Abund	Formula	Ion
724.3490	1	2061421.5	C44H45N5O5	(M+H)+
725.3522	1	1020952.1	C44H45N5O5	(M+H)+
726.3553	1	242437.1	C44H45N5O5	(M+H)+
727.3575	1	47171.6	C44H45N5O5	(M+H)+
746.3310	1	617253.4	C44H45N5O5	(M+Na)+
747.3337	1	320428.3	C44H45N5O5	(M+Na)+
748.3364	1	83406.3	C44H45N5O5	(M+Na)+
749.3389	1	17693.1	C44H45N5O5	(M+Na)+
751.3600	1	28271.3	C44H45N5O5	(M+Na)+
752.3592	1	90236.4	C44H45N5O5	(M+Na)+

--- End Of Report ---

Figure S25. LC-MS of reaction solution of **ATP-LW** (1 μM) with $ONOO^-$ (2 μM). The peak at m/z = 724.3490 indicates the conversion from boronic pinacol ester to naphthol.

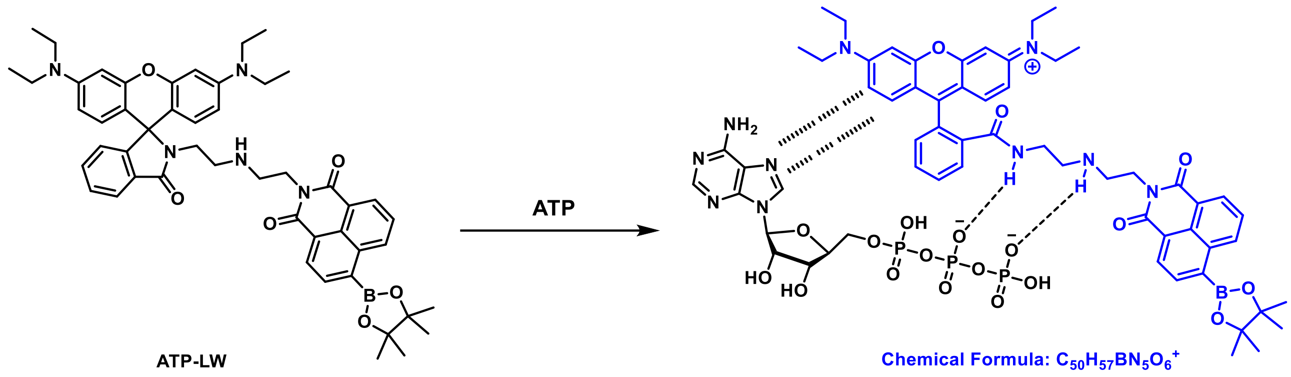

Scheme S4. Reaction mechanism between probe **ATP-LW** and ATP.

Probe **ATP-LW** (5 mM, in DMSO) was diluted to 10 μM in water, then ATP (4 mM) was added. The formation of the product highlighted in blue was determined based on Figure S26.

Walkup MS Report

**Data File**	probe +ATP_Pos_5mins_MS_15992.d	**Sample Name**	probe +ATP
**Sample Type**	Sample	**Position**	P1-A11
**Instrument Name**		**User Name**	Xue Tian
**Acq Method**	Pos_5mins_MS.m	**Acquired Time**	09/02/2021 18:01:54
**IRM Calibration Status**	Success	**DA Method**	Pos_5mins_MS.m
**Comment**			

**Sample Group**		**Info.**	
**Walkup Sample Description**		**Walkup Method**	Pos_5Mins_C18
**Formula**	C50H57BN5O6	**Walkup Method Description**	Positive mode ionization using C18 column chromatography
**Stream Name**	LC 1	**Acquisition SW Version**	6200 series TOF/6500 series Q-TOF B.09.00 (B9044.0)

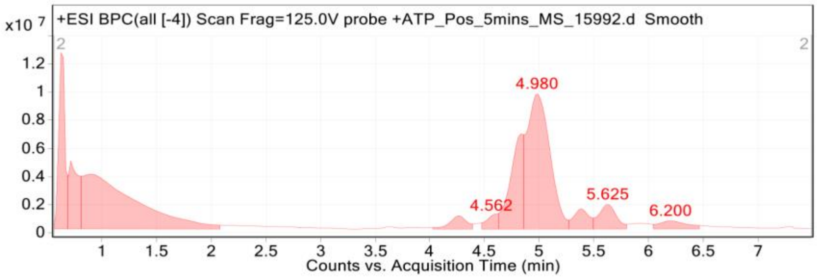

Figure 1: Base peak chromatogram

User Chromatogram Peak List

RT (min)	Area	Area %	Area Sum (%)	Base Peak (m/z)	Width (min)
0.64	50192390	36.12	10.68	508.0155	0.063
0.72	30390507	21.87	6.46	101.0066	0.104
0.91	133932251	96.37	28.49	508.0141	0.461
4.26	10136397	7.29	2.16	185.1201	0.159
4.56	7589038	5.46	1.61	696.3138	0.121
4.84	55158993	39.69	11.73	752.3784	0.123
4.98	138970659	100.00	29.56	834.4581	0.215
5.38	14060146	10.12	2.99	443.3447	0.142
5.63	19373665	13.94	4.12	666.4784	0.163
6.20	10278688	7.40	2.19	141.9629	0.266

Compound Table

Compound Label	RT (min)	Observed mass (m/z)	Neutral observed mass (Da)	Theoretical mass (Da)	Mass error (ppm)	Isotope match score (%)
Cpd 1: C50 H57 B N5 O6	4.96	834.4406	833.4420	833.4438	-2.24	99.46

Mass errors of between -5.00 and 5.00 ppm with isotope match scores above 60% are considered confirmation of molecular formulae

Walkup MS Report

Compound specific information

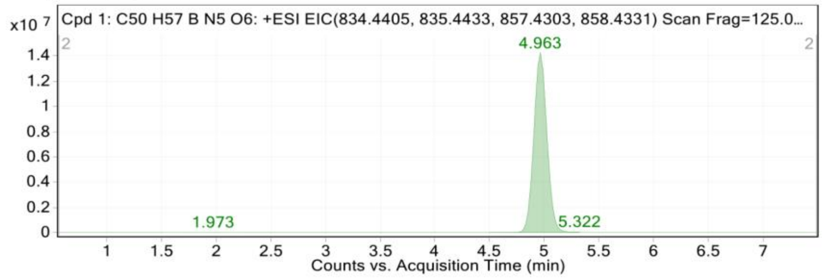

Figure: Extracted ion chromatogram (EIC) of compound.

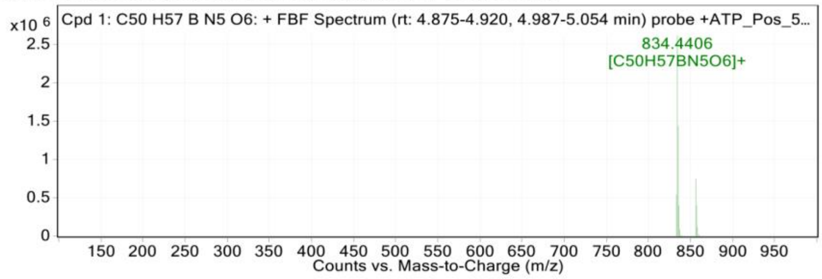

Figure: Full range view of Compound spectra and potential adducts.

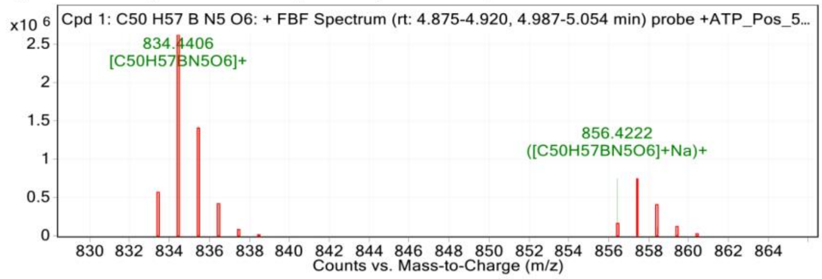

Figure: Zoomed Compound spectra view
(red boxes indicating expected theoretical isotope spacing and abundance)

Compound isotope peak List

m/z	z	Abund	Formula	Ion
833.4430	1	540591.1	C50H57BN5O6	M+
834.4406	1	2617015.5	C50H57BN5O6	M+
835.4431	1	1435080.1	C50H57BN5O6	M+
836.4458	1	399682.7	C50H57BN5O6	M+
837.4476	1	85636.1	C50H57BN5O6	M+
838.4457	1	16059.3	C50H57BN5O6	M+
856.4222	1	747684.4	C50H57BN5O6	(M+Na)+
857.4250	1	400063.3	C50H57BN5O6	(M+Na)+
858.4273	1	116222.5	C50H57BN5O6	(M+Na)+
859.4286	1	26244.7	C50H57BN5O6	(M+Na)+

--- End Of Report ---

Figure S26. LC-MS of reaction solution of **ATP-LW** (10 μM) with ATP (4 mM).

9. Protocols for cell culture

Human hepatocytes (HL-7702) were purchased from the Cell Bank of the Chinese Academy of Sciences (Shanghai, China). Cells were cultured in high-glucose DMEM supplemented with 10% fetal bovine serum, 1% penicillin and 1% streptomycin (w v^{-1}) at 37 °C in a 5% CO_2/ 95% air MCO-15AC incubator (SANYO, Tokyo, Japan).

10.MTT assay and fluorescence imaging in live cells

10.1 MTT assay

Standard 3-(4,5-dimethylthiazol-2-yl)-2,5-diphenyltetrazolium bromide (MTT) assays were carried out to evaluate the toxicity of probe **ATP-LW**. Hepatocytes (10^6 cells mL^{-1}) were seeded into 96-well microtiter plates with total volumes of 200 µL $well^{-1}$. After 12 h of incubation, various concentrations of **ATP-LW** (0 M, 1×10^{-3} M, 1×10^{-4} M, 1×10^{-5} M, 1×10^{-6} M, 1×10^{-7} M, 1×10^{-8} M and 1×10^{-9} M) were added, and the cells were cultured for another 24 h. Subsequently, an MTT solution (20 µL, 5 mg mL^{-1}, in DMEM) was added to each well. After 4 h, the MTT solution was removed, and DMSO (150 µL) was added to each well. Finally, the absorbance at 490 nm was measured using a Triturus microplate reader.

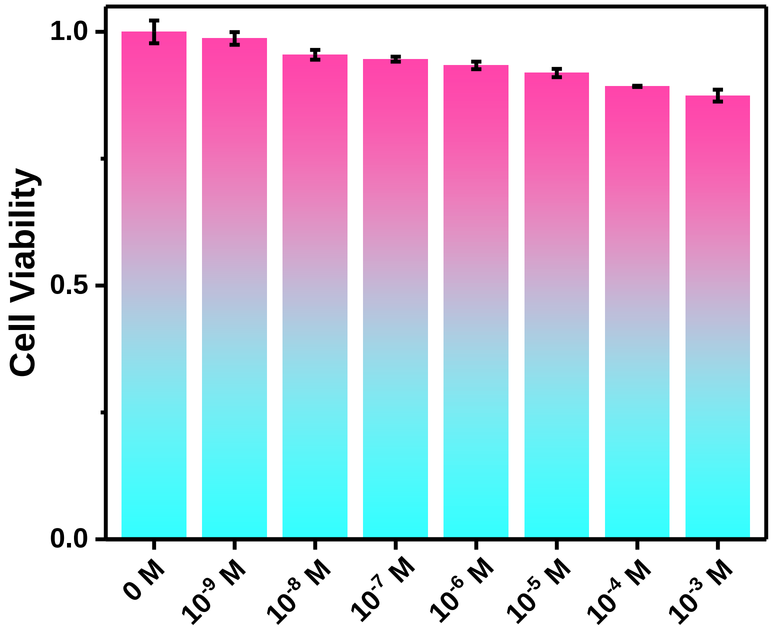

Figure S27. Cell toxicity of **ATP-LW** towards HL-7702 cells with an incubation time of 24 h. Error bar represents s.d.

10.2 One-photon fluorescence imaging of HL-7702 cells under treatment with SIN-1 or uric acid/SIN-1

Experimental procedure for Figure 4

The control group cells were stained with probe **ATP-LW** (20 µM) for 20 min. The SIN-1 stimulated group cells were incubated with SIN-1 (1 mM) for 1 h, then stained with probe **ATP-LW** (20 µM) for 20 min. The uric acid/SIN-1 stimulated group cells were incubated with uric acid (500 µM) for 1

h, followed by adding SIN-1 (1 mM) for 1 h and then stained with probe **ATP-LW** (20 μM) for 20 min. The cell culture medium of each group was removed, and all cells were washed with 1.0 mL of PBS three times before fluorescence imaging. One-photon confocal photographs were taken using a Leica SP8 high-resolution fluorescence microscope equipped with the Leica Application Suite X software package.

10.3 One-photon fluorescence imaging of HL-7702 cells under treatment with oligomycin A or oligomycin A/ATP

Experimental procedure for Figure 5

HL-7702 cells were divided into three groups. The control group cells were stained with probe **ATP-LW** (20 μM) for 20 min. The oligomycin A group cells were incubated with oligomycin A (25 μM) for 1 h, then stained with probe **ATP-LW** (20 μM) for 20 min. The oligomycin A/ATP stimulated group cells were incubated with oligomycin A (25 μM) for 1 h prior to treating with ATP (10 mM) for 1 h. They were then stained with probe **ATP-LW** (20 μM) for 20 min. The cell culture medium of each group was removed, and all cells were washed with 1.0 mL of PBS three times before fluorescence imaging. One-photon confocal photographs were taken imaged by Leica SP8 high-resolution fluorescence microscope and Leica Application Suite X software.

10.4 One-photon fluorescence imaging of HL-7702 cells under treatment with SIN-1/ATP which validates no interference for both green channel excited by 514 nm and red channel under excitation at 488 nm

Experimental procedure for Figure S28

Control group cells were stained with probe **ATP-LW** (20 μM) for 20 min. The SIN-1 + ATP group cells were incubated with SIN-1 (1 mM) for 1 h, followed by adding ATP (10 mM) for 1 h and then stained with probe **ATP-LW** (20 μM) for 20 min. The cell culture medium of each group was removed, and all cells were washed with 1.0 mL of PBS three times before fluorescence imaging. One-photon confocal photographs were taken imaged by Leica SP8 high-resolution fluorescence microscope equipped with the Leica Application Suite X software.

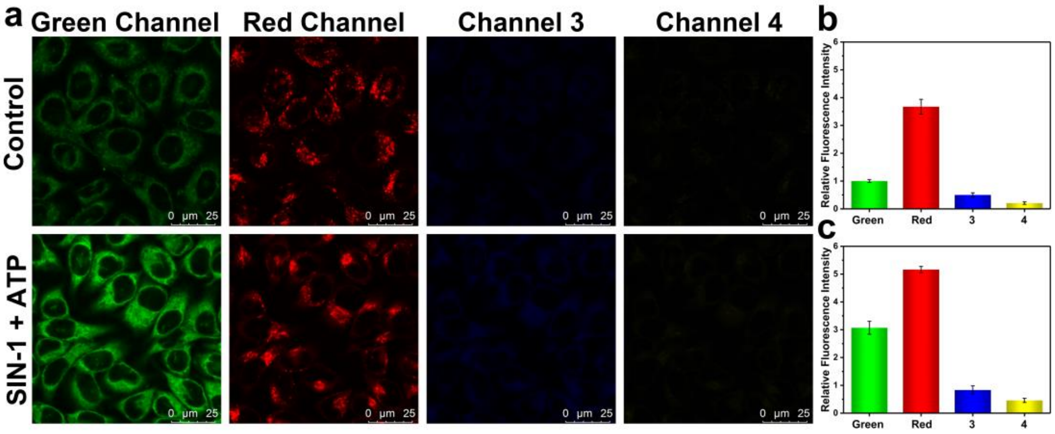

Figure S28. One-photon confocal imaging of $ONOO^-$ and ATP levels in hepatocytes. (a) One-photon fluorescence images of HL-7702 cells with the addition of SIN-1 (1 mM, 1 h) followed by addition of ATP (10 mM, 1 h) in the channels of $ONOO^-$, ATP, channel 3 and 4. Control group: Cells were stained with probe **ATP-LW** (20 μM) for 20 min. SIN-1 + ATP group: Cells were pretreated with SIN-1 (1 mM) for 1 h and followed by adding ATP (10 mM) for 1 h and then stained with probe **ATP-LW** (20 μM) for 20 min. Green fluorescence channel for $ONOO^-$: λ_{ex} = 488 nm, λ_{em} = 500-575 nm. Red fluorescence channel for ATP: λ_{ex} = 514 nm, λ_{em} = 575-650 nm. Channel 3: λ_{ex} = 488 nm, λ_{em} = 575-650 nm. Channel 4: λ_{ex} = 514 nm, λ_{em} = 520-575 nm. (b) Relative fluorescence intensity output of control group. (c) Relative fluorescence intensity output of SIN-1 + ATP group. Note: The green fluorescence intensity of control group is defined as 1.0. The data are expressed as the mean ± SD. Similar results were obtained in five independent experiments.

10.5 One-photon fluorescence imaging of APAP-induced injury in HL-7702 cells

Experimental procedure for Figure 6

HL-7702 cells were divided into three groups. The control group cells were stained with probe **ATP-LW** (20 μM) for 20 min. The model group cells were incubated with APAP (15 mM) for 2 h, then stained with probe **ATP-LW** (20 μM) for 20 min. To investigate injury remediation, HL-7702 cells were pretreated with NAC (2 mM) for 2 h and then incubated with APAP (15 mM) for 2 h, followed by staining with probe **ATP-LW** (20 μM) for 20 min. The cell culture medium of each group was removed, and all cells were washed with 1.0 mL of PBS three times before fluorescence imaging. One-photon confocal photographs were taken imaged by Leica SP8 high-resolution fluorescence microscope equipped with the Leica Application Suite X software.

10.6 One-photon fluorescence imaging of various doses of APAP-induced injury in HL-7702 cells.

Experimental procedure for Figure S29

Control group cells were stained with probe **ATP-LW** (20 μM) for 20 min. The model group cells were incubated with APAP (10, 20 mM) for 2 h, then stained with probe **ATP-LW** (20 μM) for 20 min. The cell culture medium of each group was removed, and all cells were washed with 1.0 mL of PBS three times before fluorescence imaging. One-photon confocal photographs were taken imaged by Leica SP8 high-resolution fluorescence microscope equipped with the Leica Application Suite X software.

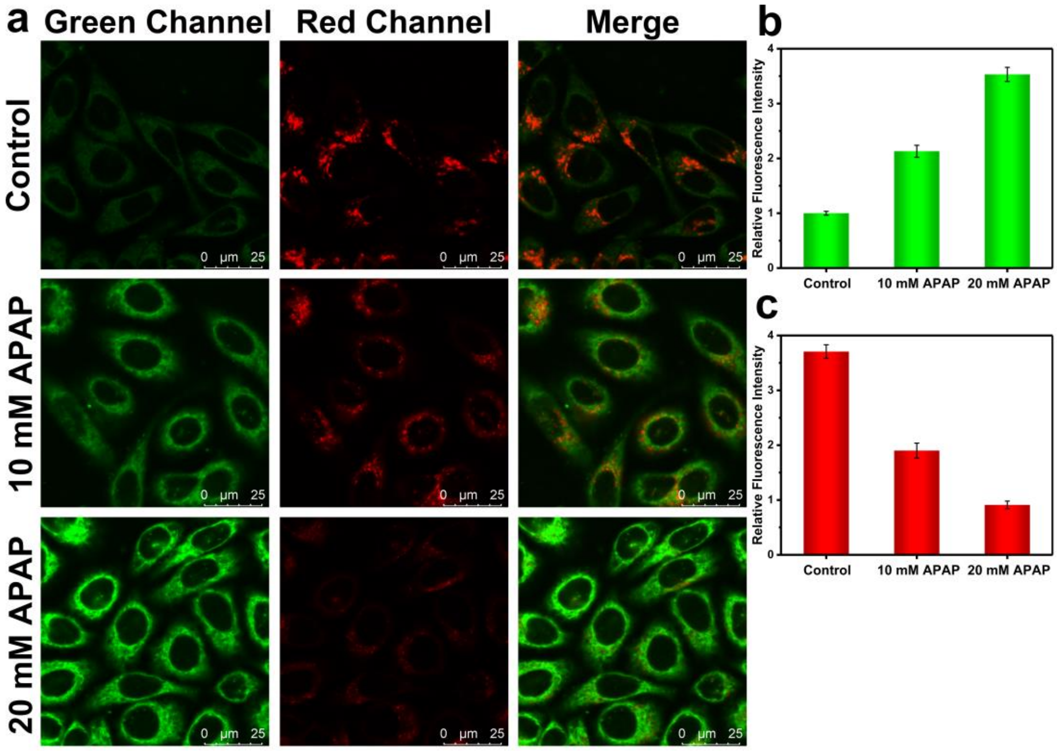

Figure S29. One-photon confocal images of various doses of APAP-induced injury with HL-7702 cells. (a) One-photon fluorescence images of HL-7702 cells with the addition of APAP (10, 20 mM, 2 h) in the channels of $ONOO^{-}$ and ATP. Control group: Cells were stained with probe **ATP-LW** (20 μM) for 20 min. 10 mM APAP group: Cells were incubated with APAP (10 mM) for 2 h, and then stained with probe **ATP-LW** (20 μM) for 20 min. 20 mM APAP group: Cells were pretreated with APAP (20 mM) for 2 h and then incubated with probe **ATP-LW** (20 μM) for another 20 min. Green fluorescence channel for $ONOO^{-}$: λ_{ex} = 488 nm, λ_{em} = 500-575 nm. Red fluorescence channel for ATP: λ_{ex} = 514 nm, λ_{em} = 575-650 nm. (b) Green relative fluorescence intensity output of three groups. (c) Red relative fluorescence intensity output of three groups. Note: The green fluorescence intensity of control group is defined as 1.0. The data are expressed as the mean ± SD. Similar results were obtained in five independent experiments.

10.7 Two-photon fluorescence imaging of APAP-induced injury in HL-7702 cells.

Experimental procedure for Figure 7

HL-7702 cells were divided into three groups. The control group cells were stained with probe **ATP-LW** (20 μM) for 20 min. The model group cells were incubated with APAP (20 mM) for 2 h, then stained with probe **ATP-LW** (20 μM) for 20 min. To investigate injury remediation, HL-7702 cells were pretreated with NAC (2 mM) for 2 h and then incubated with APAP (20 mM) for 2 h, followed by staining with probe **ATP-LW** (20 μM) for 20 min. The cell culture medium of each group was removed, and all cells were washed with 1.0 mL of PBS three times before fluorescence imaging. Two-photon photographs were taken using a Zeiss LSM 880 NLO microscope equipped with the ZEN 2 lite software.

10.8 Co-localization assay of ATP-LW.

Experimental procedure for Figure S30. HL-7702 cells were incubated with probe **ATP-LW** (20 μM) for 20 min and then incubated with the indicated commercial organelle-specific dye for 15 min.

Experimental procedure for Figure S31. HL-7702 cells were incubated with APAP (15 mM) for 2 h, and then stained with probe **ATP-LW** (20 μM) for 20 min, followed by staining with each corresponding commercial organelle-specific dye for another 15 min.

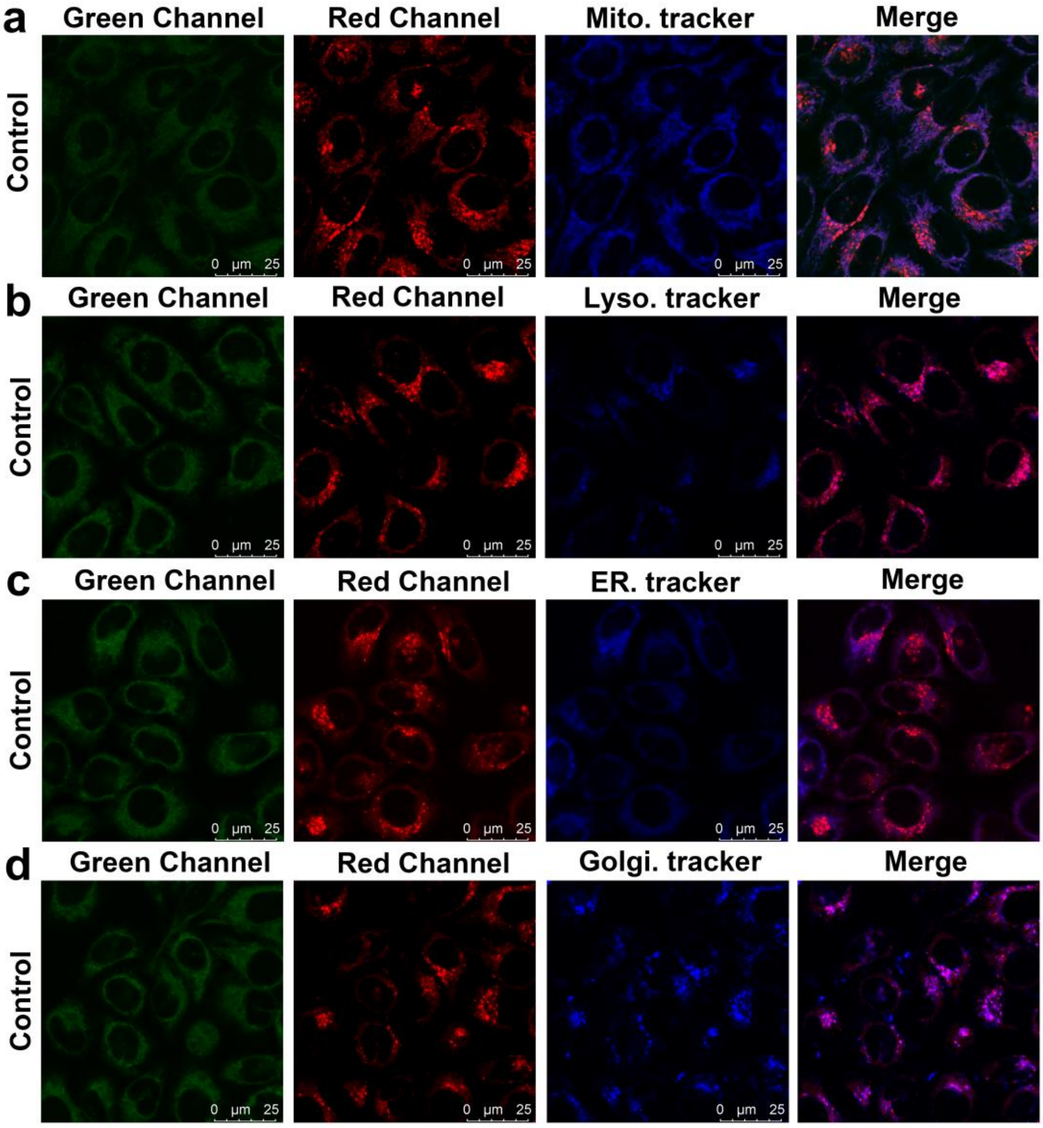

Figure S30. Confocal images of HL-7702 cells stained with probe **ATP-LW** and the indicated commercial organelle-specific dyes. (a) Fluorescence images of probe **ATP-LW** (20 μM, green channel, λ_{ex} = 488 nm, collected 500-575 nm; red channel, λ_{ex} = 514 nm, collected 575-650 nm) and Mito-Tracker® Deep Red (1 μM, blue channel, λ_{ex} = 633 nm, collected 645-763 nm) in control group cells. (b) Fluorescence images of probe **ATP-LW** (20 μM, green channel, λ_{ex} = 488 nm, collected 500-575 nm; red channel, λ_{ex} = 514 nm, collected 575-650 nm) and Lyso-Tracker® Deep Red (1 μM, blue channel, λ_{ex} = 633 nm, collected 645-763 nm) in control group cells. (c) Fluorescence images of probe **ATP-LW** (20 μM, green channel, λ_{ex} = 488 nm, collected 500-575 nm; red channel, λ_{ex} = 514 nm, collected 575-650 nm) and ER-Tracker® Red (1 μM, blue channel, λ_{ex} = 633 nm, collected 636-800 nm) in control group cells. (d) Fluorescence images of probe **ATP-LW** (20 μM, green channel, λ_{ex} = 488 nm, collected 500-575 nm; red channel, λ_{ex} = 514 nm, collected 575-650 nm) and Golgi-Tracker® Red (40 μM, blue channel, λ_{ex} = 561 nm, collected 650-700 nm) in control group cells. Merged images (a), (b), (c), (d) are overlays of the red channel of probe **ATP-LW** and the blue channel of each commercial organelle-specific dye. **The Pearson's correlation for mitochondria, lysosomes, endoplasmic reticulum and Golgi apparatus corresponding to these studies are 0.6932, 0.7686, 0.5728 and 0.6446, respectively.**

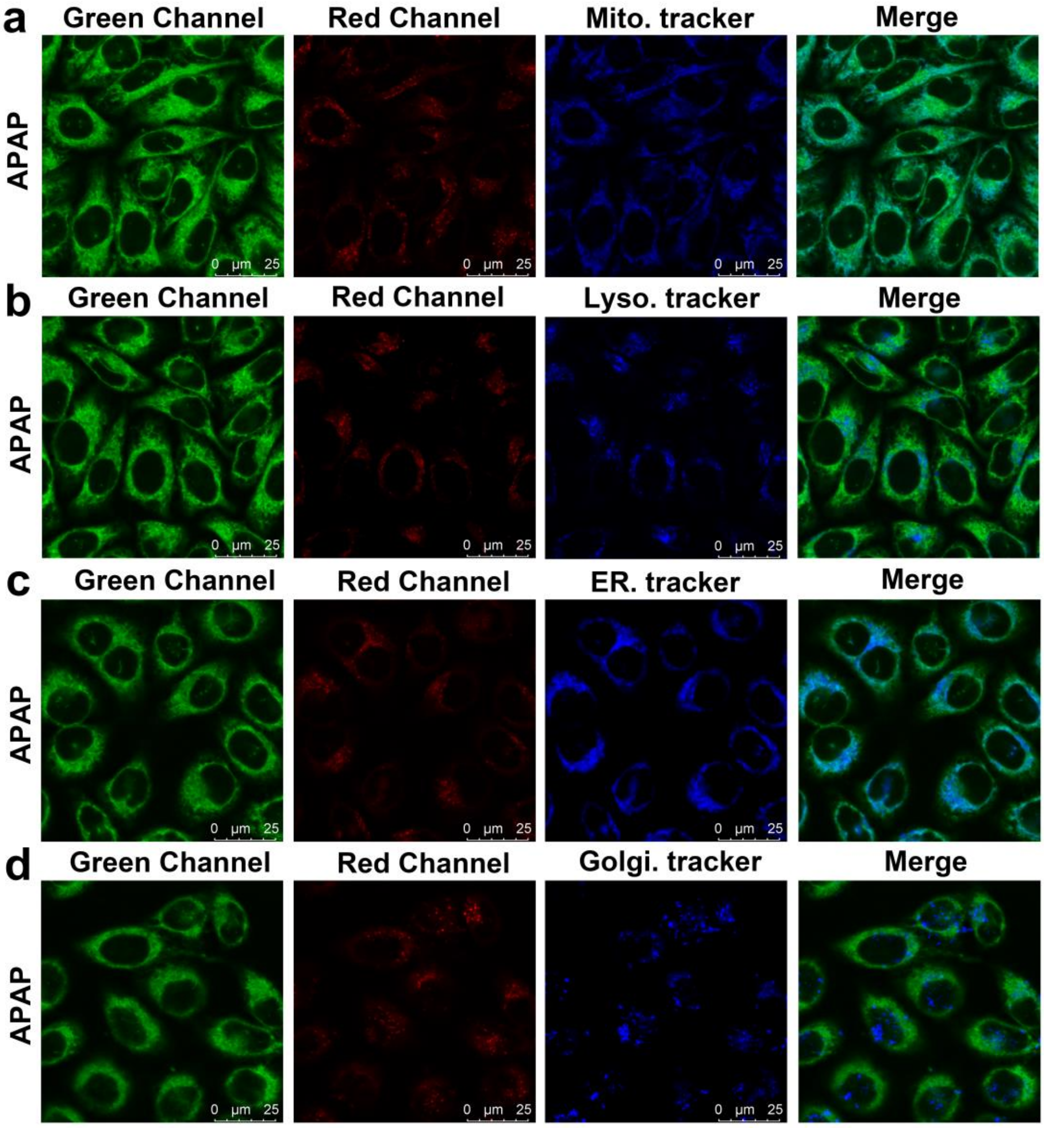

Figure S31. Confocal images of APAP HL-7702 cells stained with probe **ATP-LW** and the indicated commercial organelle-specific dyes. (a) Fluorescence images of probe **ATP-LW** (20 μM, green channel, λ_{ex} = 488 nm, collected 500-575 nm; red channel, λ_{ex} = 514 nm, collected 575-650 nm) and Mito-Tracker® Deep Red (1 μM, blue channel, λ_{ex} = 633 nm, collected 645-763 nm) in APAP (15 mM, 2 h) pretreated cells. (b) Fluorescence images of probe **ATP-LW** (20 μM, green channel, λ_{ex} = 488 nm, collected 500-575 nm; red channel, λ_{ex} = 514 nm, collected 575-650 nm) and Lyso-Tracker® Deep Red (1 μM, blue channel, λ_{ex} = 633 nm, collected 645-763 nm) in APAP (15 mM, 2 h) pretreated cells. (c) Fluorescence images of probe **ATP-LW** (20 μM, green channel, λ_{ex} = 488 nm, collected 500-575 nm; red channel, λ_{ex} = 514 nm, collected 575-650 nm) and ER-Tracker® Red (1 μM, blue channel, λ_{ex} = 633 nm, collected 636-800 nm) in APAP (15 mM, 2 h) pretreated cells. (d) Fluorescence images of probe **ATP-LW** (20 μM, green channel, λ_{ex} = 488 nm, collected 500-575 nm; red channel, λ_{ex} = 514 nm, collected 575-650 nm) and Golgi-Tracker® Red (40 μM, blue channel, λ_{ex} = 561 nm, collected 650-700 nm) in APAP (15 mM, 2 h) pretreated cells. Merge images of (a), (b), (c), (d) are overlay of green channel of probe **ATP-LW** and blue channel of commercial organelle-specific dyes, respectively. **The Pearson's correlation for the mitochondria, lysosomes, endoplasmic reticulum and Golgi apparatus corresponding to these studies are 0.8727, 0.5054, 0.7087 and 0.2858, respectively.**

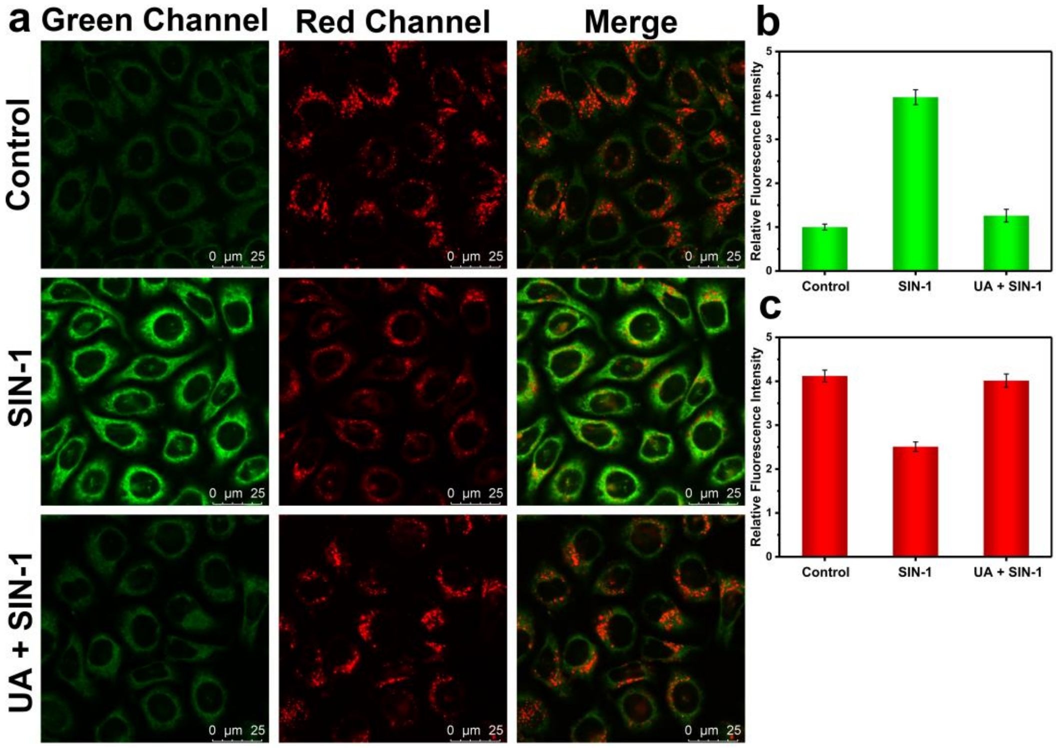

Figure S32. Original image for **Figure 4**: One-photon confocal imaging of $ONOO^-$ and ATP levels in hepatocytes treated with SIN-1 or SIN-1/uric acid. (a) One-photon fluorescence images of HL-7702 cells recorded after the addition of SIN-1 (1 mM, 1 h) and uric acid (500 μM, 1 h) with monitoring over the green ($ONOO^-$) and red (ATP) channels. Control group: Cells were stained with probe **ATP-LW** (20 μM) for 20 min. SIN-1 group: cells were incubated with SIN-1 (1 mM) for 1 h, then stained with probe **ATP-LW** (20 μM) for 20 min. UA + SIN-1 group: Cells were pretreated with uric acid (500 μM) for 1 h and followed by adding SIN-1 (1 mM) for 1 h and then stained with probe **ATP-LW** (20 μM) for 20 min. Green fluorescence channel for $ONOO^-$: λ_{ex} = 488 nm, λ_{em} = 500-575 nm. Red fluorescence channel for ATP: λ_{ex} = 514 nm, λ_{em} = 575-650 nm. (b) Green relative fluorescence intensity output of three groups. (c) Red relative fluorescence intensity output of three groups. Note: The green fluorescence intensity of the control group is defined as 1.0. The data are expressed as the mean ± SD. Concordant results were obtained from five independent experiments.

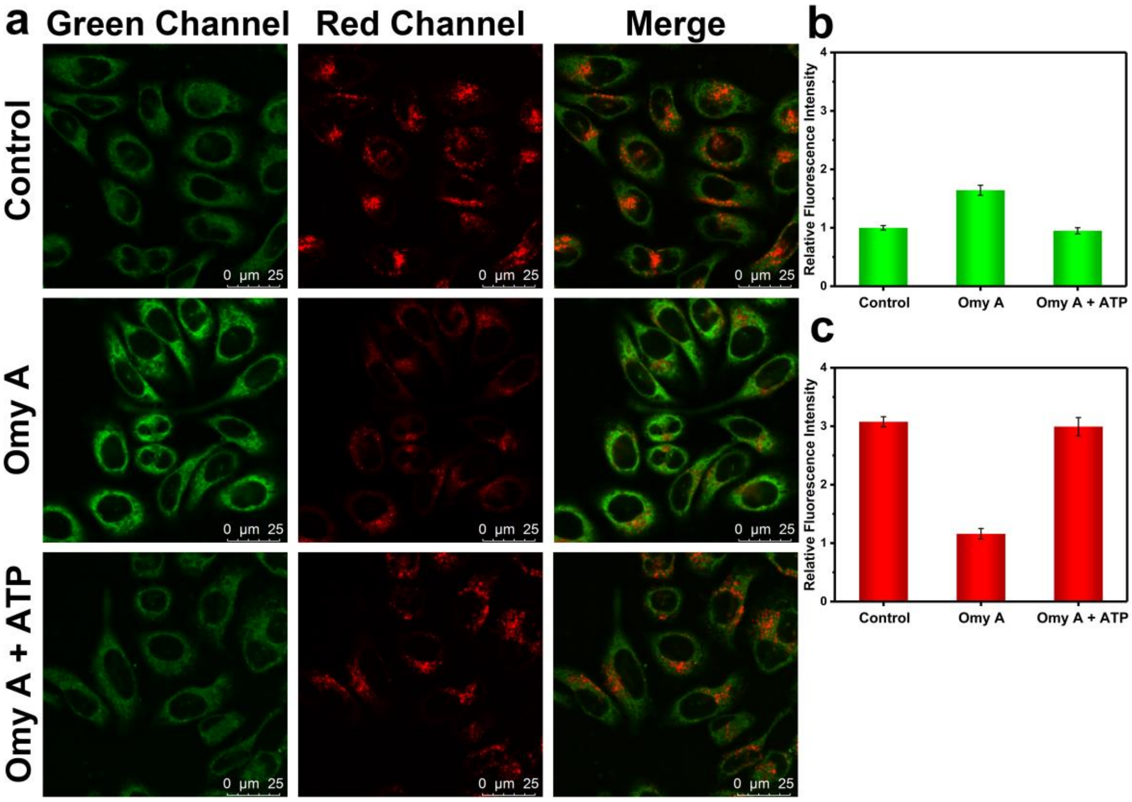

Figure S33. Original image for **Figure 5**: One-photon confocal imaging of $ONOO^-$ and ATP levels in hepatocytes treated with omy A or omy A/ATP. (a) One-photon fluorescence images of HL-7702 cells with the addition of omy A (25 μM, 1 h) and ATP (10 mM, 1 h) for green ($ONOO^-$) and red (ATP) channels. Control group: Cells were stained with probe **ATP-LW** (20 μM) for 20 min. Omy A group: Cells were incubated with omy A (25 μM) for 1 h, then stained with probe **ATP-LW** (20 μM) for 20 min. Omy A + ATP group: Cells were pretreated with omy A (25 μM) for 1 h and followed by adding ATP (10 mM) for 1 h and then stained with probe **ATP-LW** (20 μM) for 20 min. Green fluorescence channel for $ONOO^-$: λ_{ex} = 488 nm, λ_{em} = 500-575 nm. Red fluorescence channel for ATP: λ_{ex} = 514 nm, λ_{em} = 575-650 nm. (b) Green relative fluorescence intensity output of three groups. (c) Red relative fluorescence intensity output of three groups. Note: The green fluorescence intensity of the control group is defined as 1.0. The data are expressed as the mean ± SD. Concordant results were obtained from five independent experiments.

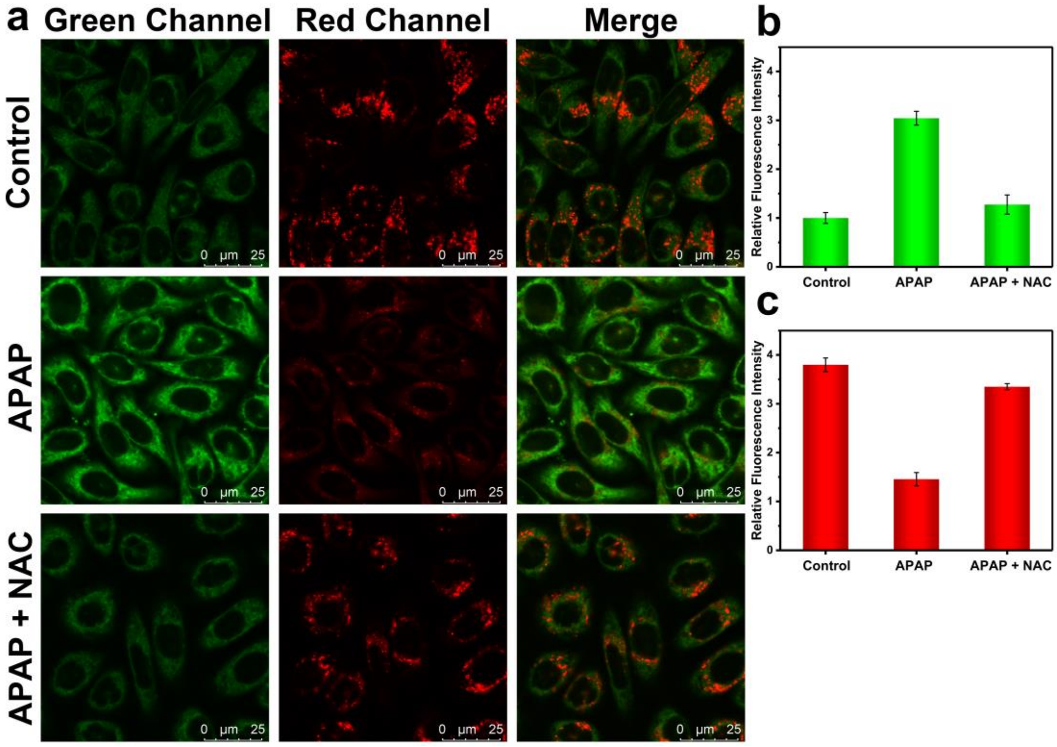

Figure S34. Original image for **Figure 6**: One-photon confocal images of APAP-induced injury and its remediation by NAC in HL-7702 cells. (a) One-photon fluorescence images of HL-7702 cells with the addition of APAP (15 mM, 2 h) and NAC (2 mM, 2 h) for green ($ONOO^-$) and red (ATP) channels. Control group: Cells were stained with probe **ATP-LW** (20 µM) for 20 min. APAP group: Cells were incubated with APAP (15 mM) for 2 h, and then stained with probe **ATP-LW** (20 µM) for 20 min. APAP + NAC group: Cells were pretreated with NAC (2 mM) for 2 h and then incubated with APAP (15 mM) for 2 h, followed by staining with probe **ATP-LW** (20 µM) for another 20 min. Green fluorescence channel for $ONOO^-$: λ_{ex} = 488 nm, λ_{em} = 500-575 nm. Red fluorescence channel for ATP: λ_{ex} = 514 nm, λ_{em} = 575-650 nm. (b) Green relative fluorescence intensity output of three groups. (c) Red relative fluorescence intensity output of three groups. Note: The green fluorescence intensity of the control group is defined as 1.0. The data are expressed as the mean ± SD. Concordant results were obtained from five independent experiments.

11.NMR spectra

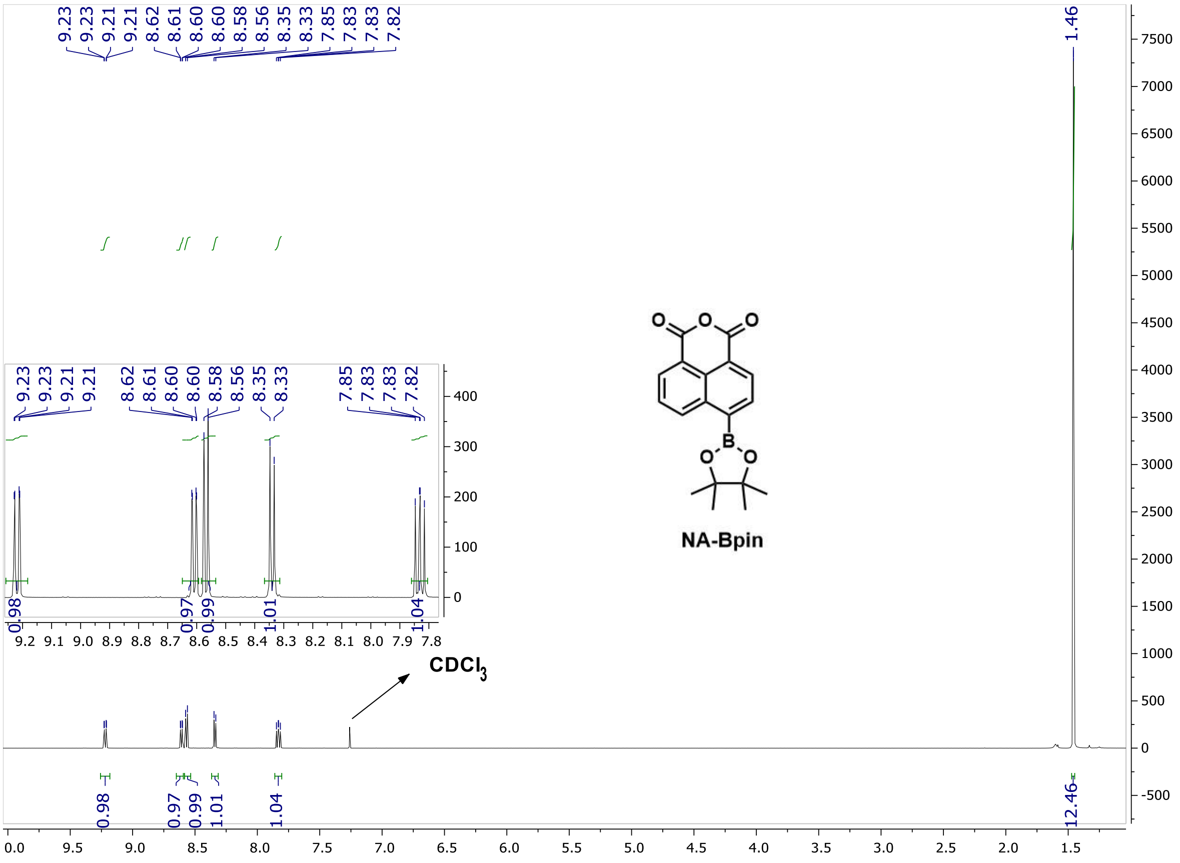

Figure S35. ^{1}H NMR (500 MHz, $CDCl_3$) of **NA-Bpin**.

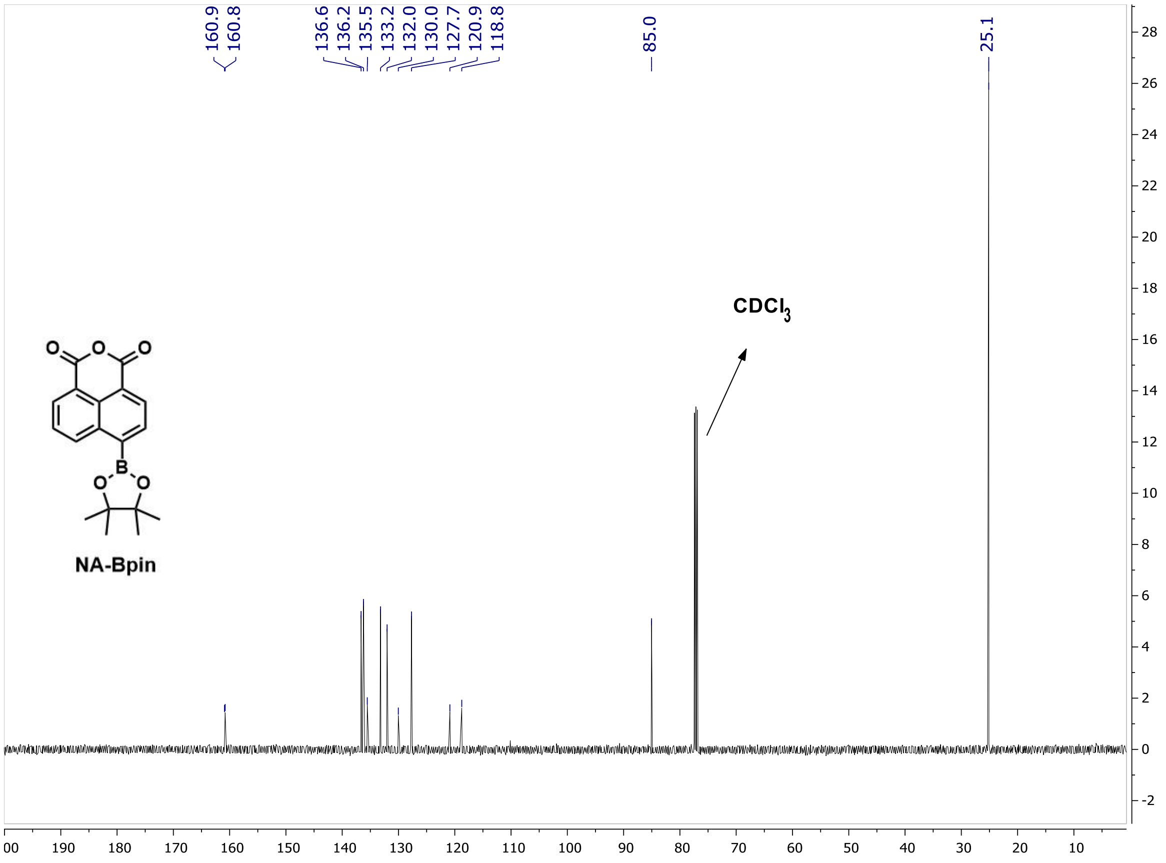

Figure S36. ^{13}C NMR (126 MHz, $CDCl_3$) of **NA-Bpin**.

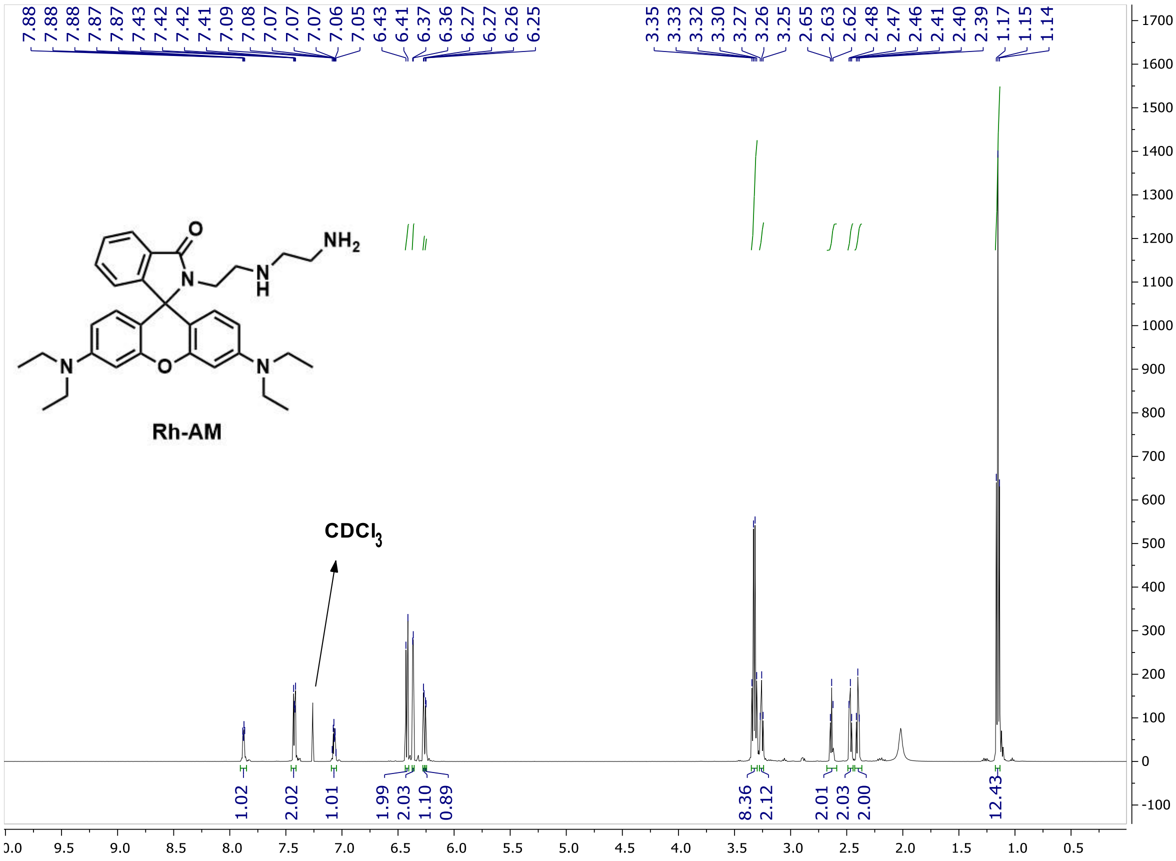

Figure S37. ^{1}H NMR (500 MHz, $CDCl_3$) of **Rh-AM**.

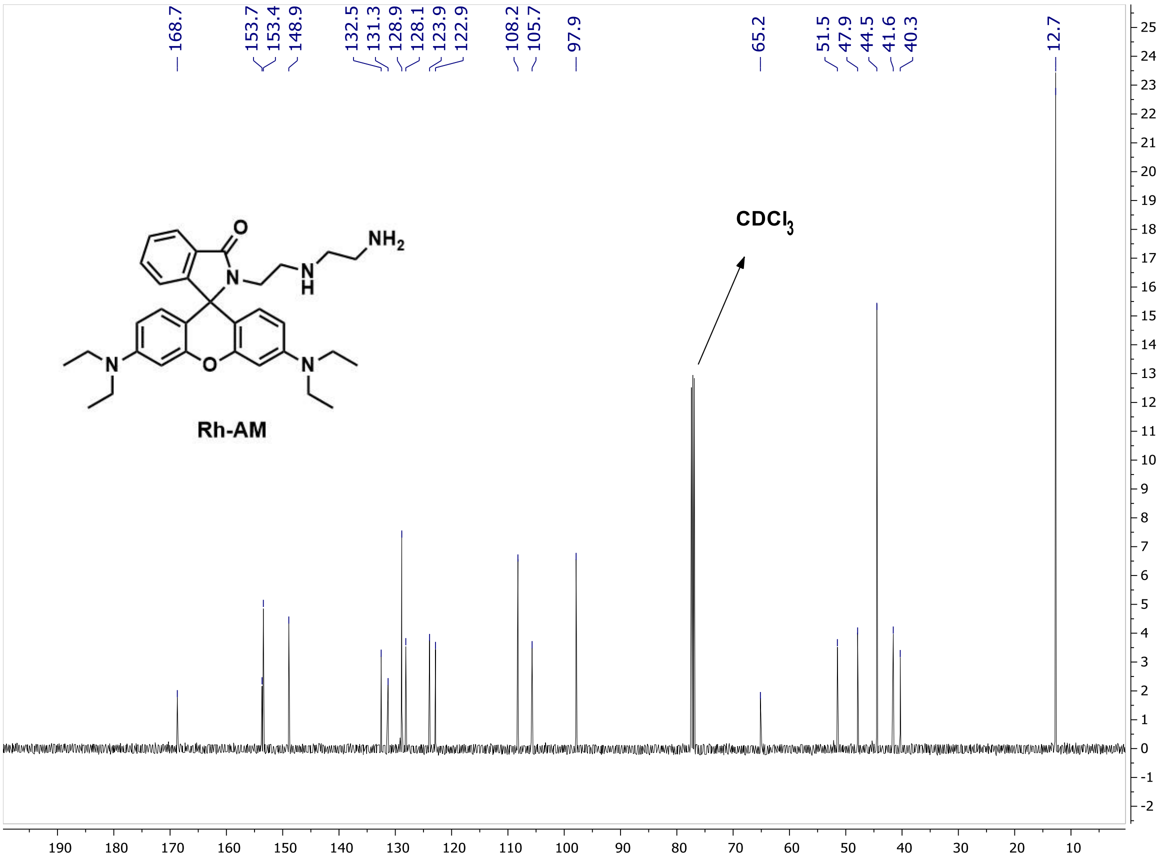

Figure S38. ^{13}C NMR (126 MHz, $CDCl_3$) of **Rh-AM**.

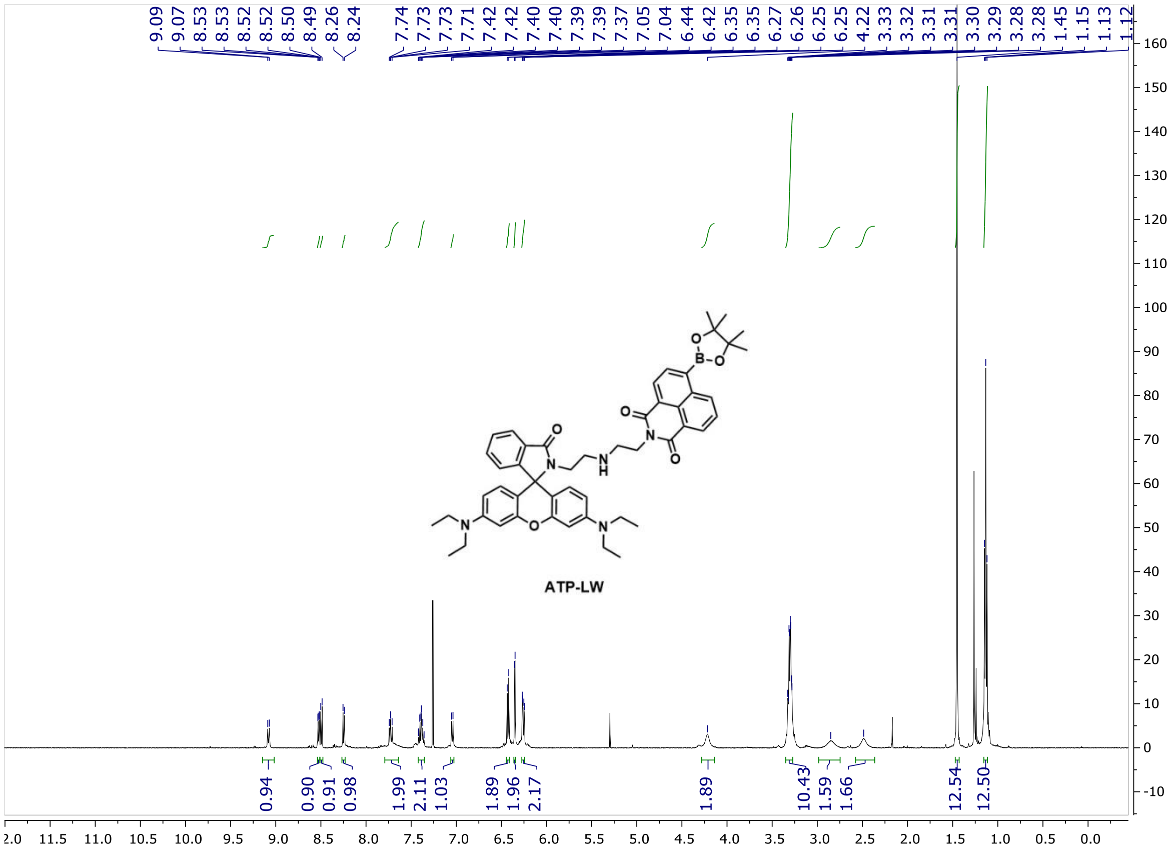

Figure S39. ^{1}H NMR (500 MHz, $CDCl_3$) of **ATP-LW**.

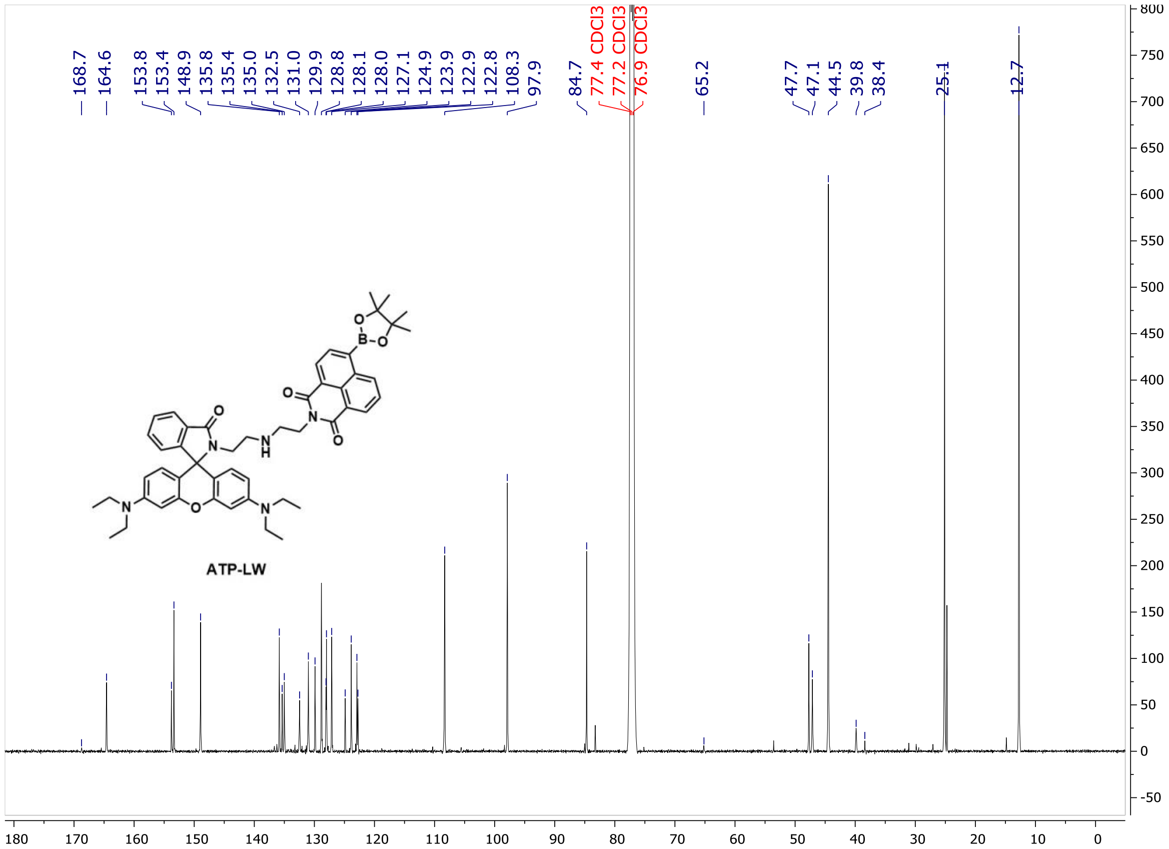

Figure S40. ^{13}C NMR (126 MHz, $CDCl_3$) of **ATP-LW**.

12.Author contributions

Luling Wu – Designed the probe with Chusen Huang, synthesized the probe, designed the solution and cell experiments, drafted the manuscript/ESI and helped respond to the referees' comments.
Jihong Liu – Carried out all the biological work under supervision of Ping Li and Bo Tang.
Xue Tian – Carried out all the solution work.
Robin R. Groleau – Carried out and advised on NMR characterization and reviewed the manuscript.
Beidong Feng – Performed the transient absorption spectral experiments under the supervision of Hua Zhang
Yonggang Yang, Hua Zhang – Carried out the theoretical calculations
Hai-Hao Han, Han-Min Wang, Yi Zang, Jia Li, and Xiao-Peng He – Carried out cellular experiments; however, these data are not added in the manuscript/ESI since it was found that only exogenous stimuli were effective in imaging fluctuations in ATP/$ONOO^-$ concentrations.
Yang Wang – Synthesized a related probe that was ultimately not included in the manuscript/ESI.
Chusen Huang – Supervisor of Yang Wang who designed the probe in conjunction with Luling Wu
Fang Huang – Carried out preliminary theoretical calculations prior to this task being taken over by the group of Hua Zhang.
Steven D. Bull – Supervisor of Robin R. Groleau, Luling Wu and Xue Tian
Tony D. James – Lead supervisor, reviewed and edited the manuscript/ESI
Jonathan L. Sessler – Supervisor of Adam C. Sedgwick and reviewed the manuscript/ESI with Adam C. Sedgwick; helped finalize both documents.

13.References

1. Manyike, P. T.; Kharasch, E. D.; Kalhorn, T. F.; Slattery, J. T., Contribution of CYP2E1 and CYP3A to acetaminophen reactive metabolite formation. *Clin. Pharmacol. Ther.* **2000,** *67*, 275-282.
2. Chen, W.; Koenigs, L. L.; Thompson, S. J.; Peter, R. M.; Rettie, A. E.; Trager, W. F.; Nelson, S. D., Oxidation of acetaminophen to its toxic quinone imine and nontoxic catechol metabolites by baculovirus-expressed and purified human cytochromes P450 2E1 and 2A6. *Chem. Res. Toxicol.* **1998,** *11*, 295-301.
3. Hinson, J. A.; Roberts, D. W.; James, L. P., Mechanisms of acetaminophen-induced liver necrosis. In *Adverse Drug Reactions*, Uetrecht, J., Ed. Springer Berlin Heidelberg: Berlin, Heidelberg, **2010**; pp 369-405.
4. Chen, J.; Huang, D.; She, M.; Wang, Z.; Chen, X.; Liu, P.; Zhang, S.; Li, J., Recent progress in fluorescent sensors for drug-induced liver injury assessment. *ACS Sens.* **2021,** *6*, 628-640.
5. Lee, K. K.; Imaizumi, N.; Chamberland, S. R.; Alder, N. N.; Boelsterli, U. A., Targeting mitochondria with methylene blue protects mice against acetaminophen-induced liver injury. *Hepatology* **2015,** *61*, 326-336.
6. Du, K.; Ramachandran, A.; Weemhoff, J. L.; Chavan, H.; Xie, Y.; Krishnamurthy, P.; Jaeschke, H., Editor's highlight: metformin protects against acetaminophen hepatotoxicity by attenuation of mitochondrial oxidant stress and dysfunction. *Toxicol. Sci.* **2016,** *154*, 214-226.
7. Jaeschke, H.; McGill, M. R.; Ramachandran, A., Oxidant stress, mitochondria, and cell death mechanisms in drug-induced liver injury: Lessons learned from acetaminophen hepatotoxicity. *Drug Metab. Rev.* **2012,** *44*, 88-106.
8. Wu, L.; Han, H.-H.; Liu, L.; Gardiner, J. E.; Sedgwick, A. C.; Huang, C.; Bull, S. D.; He, X.-P.; James, T. D., ESIPT-based fluorescence probe for the rapid detection of peroxynitrite 'AND' biological thiols. *Chem. Comm.* **2018,** *54*, 11336-11339.
9. Rushworth, G. F.; Megson, I. L., Existing and potential therapeutic uses for N-acetylcysteine: The need for conversion to intracellular glutathione for antioxidant benefits. *Pharmacol. Ther.* **2014,** *141*, 150-159.
10. Armstrong, J. S.; Whiteman, M.; Rose, P.; Jones, D. P., The coenzyme Q_{10} analog decylubiquinone inhibits the redox-activated mitochondrial permeability transition: role of mitcohondrial respiratory complex III. *J. Biol. Chem.* **2003,** *278*, 49079-49084.
11. Briston, T.; Roberts, M.; Lewis, S.; Powney, B.; M. Staddon, J.; Szabadkai, G.; Duchen, M. R., Mitochondrial permeability transition pore: sensitivity to opening and mechanistic dependence on substrate availability. *Sci. Rep.* **2017,** *7*, 10492.
12. Lee, S. J.; Jin, Y.; Yoon, H. Y.; Choi, B.-O.; Kim, H. C.; Oh, Y.-K.; Kim, H.-S.; Kim, W.-K., Ciclopirox protects mitochondria from hydrogen peroxide toxicity. *Br. J. Pharmacol.* **2005,** *145*, 469-476.
13. Radi, R.; Cassina, A.; Hodara, R.; Quijano, C.; Castro, L., Peroxynitrite reactions and formation in mitochondria. *Free Radical Biol. Med.* **2002,** *33*, 1451-1464.
14. Hunter, D. R.; Haworth, R. A., The Ca^{2+}-induced membrane transition in mitochondria: I. The protective mechanisms. *Arch. Biochem. Biophys.* **1979,** *195*, 453-459.
15. Bonora, M.; Pinton, P., The mitochondrial permeability transition pore and cancer: molecular mechanisms involved in cell death. *Front. Oncol.* **2014,** *4*, 302.
16. White, R. J.; Reynolds, I. J., Mitochondrial depolarization in glutamate-stimulated neurons: an early signal specific to excitotoxin exposure. *J. Neurosci.* **1996,** *16*, 5688-5697.
17. Okuno, D.; Iino, R.; Noji, H., Rotation and structure of F_oF_1-ATP synthase. *J. Biochem.* **2011,** *149*, 655-664.
18. Diez, M.; Zimmermann, B.; Börsch, M.; König, M.; Schweinberger, E.; Steigmiller, S.; Reuter, R.; Felekyan, S.; Kudryavtsev, V.; Seidel, C. A. M.; Gräber, P., Proton-powered subunit rotation in single membrane-bound F_oF_1-ATP synthase. *Nat. Struct. Mol. Biol.* **2004,** *11*, 135-141.
19. Moles, A.; Torres, S.; Baulies, A.; Garcia-Ruiz, C.; Fernandez-Checa, J. C., Mitochondrial–lysosomal axis in

acetaminophen hepatotoxicity. *Front. Pharmacol.* **2018,** *9*, 453.

20. Liang, X.; Xu, X.; Qiao, D.; Yin, Z.; Shang, L., Dual mechanism of an intramolecular charge transfer (ICT)–FRET-based fluorescent probe for the selective detection of hydrogen peroxide. *Chem. Asian J.* **2017,** *12*, 3187-3194.
21. Bag, B.; Biswal, B., Alteration of selectivity in rhodamine based probes for Fe(iii) and Hg(ii) ion induced dual mode signalling responses. *Org. Biomol. Chem.* **2012,** *10*, 2733-2738.
22. Fang, Y.; Shi, W.; Hu, Y.; Li, X.; Ma, H., A dual-function fluorescent probe for monitoring the degrees of hypoxia in living cells via the imaging of nitroreductase and adenosine triphosphate. *Chem. Comm.* **2018,** *54*, 5454-5457.
23. Frisch, M. J.; Trucks, G. W.; Schlegel, H. B.; Scuseria, G. E.; Robb, M. A.; Cheeseman, J. R.; Scalmani, G.; Barone, V.; Petersson, G. A.; Nakatsuji, H.; Li, X.; Caricato, M.; Marenich, A. V.; Bloino, J.; Janesko, B. G.; Gomperts, R.; Mennucci, B.; Hratchian, H. P.; Ortiz, J. V.; Izmaylov, A. F.; Sonnenberg, J. L.; Williams-Young, D.; Ding, F.; Lipparini, F.; Egidi, F.; Goings, J.; Peng, B.; Petrone, A.; Henderson, T.; Ranasinghe, D.; Zakrzewski, V. G.; Gao, J.; Rega, N.; Zheng, G.; Liang, W.; Hada, M.; Ehara, M.; Toyota, K.; Fukuda, R.; Hasegawa, J.; Ishida, M.; Nakajima, T.; Honda, Y.; Kitao, O.; Nakai, H.; Vreven, T.; Throssell, K.; Montgomery, J. A., Jr.; Peralta, J. E.; Ogliaro, F.; Bearpark, M. J.; Heyd, J. J.; Brothers, E. N.; Kudin, K. N.; Staroverov, V. N.; Keith, T. A.; Kobayashi, R.; Normand, J.; Raghavachari, K.; Rendell, A. P.; Burant, J. C.; Iyengar, S. S.; Tomasi, J.; Cossi, M.; Millam, J. M.; Klene, M.; Adamo, C.; Cammi, R.; Ochterski, J. W.; Martin, R. L.; Morokuma, K.; Farkas, O.; Foresman, J. B.; Fox, D. J., Gaussian 16, Revision B.01, Gaussian, Inc., Wallingford CT. **2016**.
24. Zhao, Y.; Truhlar, D. G., The M06 suite of density functionals for main group thermochemistry, thermochemical kinetics, noncovalent interactions, excited states, and transition elements: two new functionals and systematic testing of four M06-class functionals and 12 other functionals. *Theor. Chem. Acc.* **2008,** *120*, 215-241.
25. Schäfer, A.; Huber, C.; Ahlrichs, R., Fully optimized contracted Gaussian basis sets of triple zeta valence quality for atoms Li to Kr. *J. Chem. Phys.* **1994,** *100*, 5829-5835.
26. Weigend, F.; Ahlrichs, R., Balanced basis sets of split valence, triple zeta valence and quadruple zeta valence quality for H to Rn: Design and assessment of accuracy. *Phys. Chem. Chem. Phys.* **2005,** *7*, 3297-3305.
27. Chai, J.-D.; Head-Gordon, M., Long-range corrected hybrid density functionals with damped atom–atom dispersion corrections. *Phys. Chem. Chem. Phys.* **2008,** *10*, 6615-6620.
28. Takano, Y.; Houk, K. N., Benchmarking the conductor-like polarizable continuum model (CPCM) for aqueous solvation free energies of neutral and ionic organic molecules. *J. Chem. Theory Comput.* **2005,** *1*, 70-77.
